# Investigating the potential anticancer activities of antibiotics as topoisomerase II inhibitors and DNA intercalators: *in vitro*, molecular docking, molecular dynamics, and SAR studies

**DOI:** 10.1080/14756366.2023.2171029

**Published:** 2023-01-26

**Authors:** Faten Farouk, Ayman Abo Elmaaty, Ahmed Elkamhawy, Haytham O. Tawfik, Radwan Alnajjar, Mohammed A. S. Abourehab, Mohamed A. Saleh, Wagdy M. Eldehna, Ahmed A. Al‐Karmalawy

**Affiliations:** a Pharmaceutical Chemistry Department, Faculty of Pharmacy, Ahram Canadian University, Giza, Egypt; b Department of Medicinal Chemistry, Faculty of Pharmacy, Port Said University, Port Said, Egypt; c BK21 FOUR Team and Integrated Research Institute for Drug Development, College of Pharmacy, Dongguk University-Seoul, Goyang, Republic of Korea; d Department of Pharmaceutical Organic Chemistry, Faculty of Pharmacy, Mansoura University, Mansoura, Egypt; e Department of Pharmaceutical Chemistry, Faculty of Pharmacy, Tanta University, Tanta, Egypt; f Department of Chemistry, Faculty of Science, University of Benghazi, Benghazi, Libya; g PharmD, Faculty of Pharmacy, Libyan International Medical University, Benghazi, Libya; h Department of Chemistry, University of Cape Town, Rondebosch, South Africa; i Department of Pharmaceutics, Faculty of Pharmacy, Umm Al-Qura University, Makkah, Saudi Arabia; j Department of Clinical Sciences, College of Medicine, University of Sharjah, Sharjah, the United Arab Emirates; k Department of Pharmacology and Toxicology, Faculty of Pharmacy, Mansoura University, Mansoura, Egypt; l Department of Pharmaceutical Chemistry, Faculty of Pharmacy, Kafrelsheikh University, Kafrelsheikh, Egypt; m School of Biotechnology, Badr University in Cairo, Badr City, Egypt

**Keywords:** Antibiotics, cytotoxicity, topoisomerase II, molecular docking and dynamics, MM-GBSA

## Abstract

Topoisomerase II (TOP-2) is a promising molecular target for cancer therapy. Numerous antibiotics could interact with biologically relevant macromolecules and provoke antitumor potential. Herein, molecular docking studies were used to investigate the binding interactions of 138 antibiotics against the human topoisomerase II-DNA complex. Followed by the MD simulations for 200 ns and MM-GBSA calculations. On the other hand, the antitumor activities of the most promising candidates were investigated against three cancer cell lines using doxorubicin (DOX) as a reference drug. Notably, spiramycin (**SP**) and clarithromycin (**CL**) showed promising anticancer potentials on the MCF-7 cell line. Moreover, azithromycin (**AZ**) and **CL** exhibited good anticancer potentials against the HCT-116 cell line. Finally, the TOP-2 enzyme inhibition assay was carried out to confirm the proposed rationale. Briefly, potent TOP-2 inhibitory potentials were recorded for erythromycin (**ER**) and roxithromycin (**RO**). Additionally, a SAR study opened eyes to promising anticancer pharmacophores encountered by these antibiotics.

**Highlights**
- Molecular docking studies of 139 antibiotics against the topoisomerase II-DNA complex.
- **SP**, **RO**, **AZ**, **CL**, and **ER** were the most promising and commercially available candidates.
- Molecular dynamics simulations for 200 ns for the most promising five complexes.
- MM-GBSA calculations for the frontier five complexes.
- **SP** and **CL** showed promising anticancer potentials on the MCF-7 cell line, besides, **AZ** and **CL** exhibited good anticancer potentials against the HCT-116 cell line.
- Potent TOP-2 inhibitory potentials were recorded for **ER** and **RO**.

## Graphical Abstract

**Figure:**
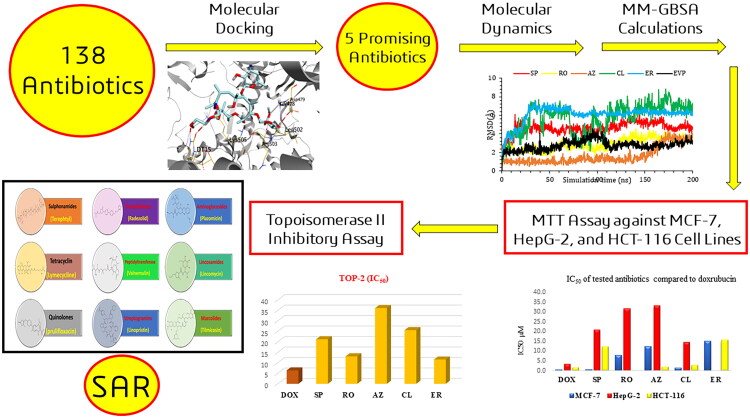

## Introduction

Numerous patients worldwide suffer from variable types of cancer and the prevalence of cancer is increasing^1^. For instance, according to GLOBOCAN 2020, the global number of cancer cases was 19 292 789 while death cases attributed to cancer were 9 958 133^2^^,^^3^. Numerous factors contribute to this, such as increased pollution and exposure to ultraviolet radiation^4^. Additionally, change in population age structure and the rise of bad lifestyle habits such as smoking, alcohol consumption, being overweight, obesity, physical inactivity, and malnutrition all contribute to the increased global prevalence of cancer^5^^,^^6^. Therefore, the development of cancer therapy is an ongoing research quest. In the past, cancer therapy relied on surgeries and radiotherapy. Currently, molecular cancer cell targets are the main approach for the development of an anticancer agent^7^^,^^8^.

Topoisomerase II (TOP-2) is a promising molecular target for cancer therapy. It is a nuclear enzyme that is essential for cell survival. TOP-2 is involved in various stages of DNA replication as well as chromosome assembly and segregation. It acts by breaking the double-stranded DNA and promoting the relaxation of over-coiled strands^9^^,^^10^. Therefore, it is responsible for the topological changes in double-stranded DNA that are required prior to replication and transcription. TOP-2 exerts its function *via* introducing a protein-bridged cut in DNA strands^11^. A higher level of TOP-2 expression and a prolonged intracellular half-life is observed in malignant cells over normal ones making the enzyme a potential target for anticancer therapy. Drug candidates that target TOP-2 can be classified as TOP-2 poisons and TOP-2 catalytic inhibitors. The TOP-2 poisons exert their function by increasing the covalent binding between the TOP-2 and DNA. This group includes most of the clinically relevant compounds e.g. doxorubicin (DOX). The TOP-2 catalytic inhibitors act by halting the activity of TOP-2 which is crucial for tumour survival^12^^,^^13^.

**Figure 1.**
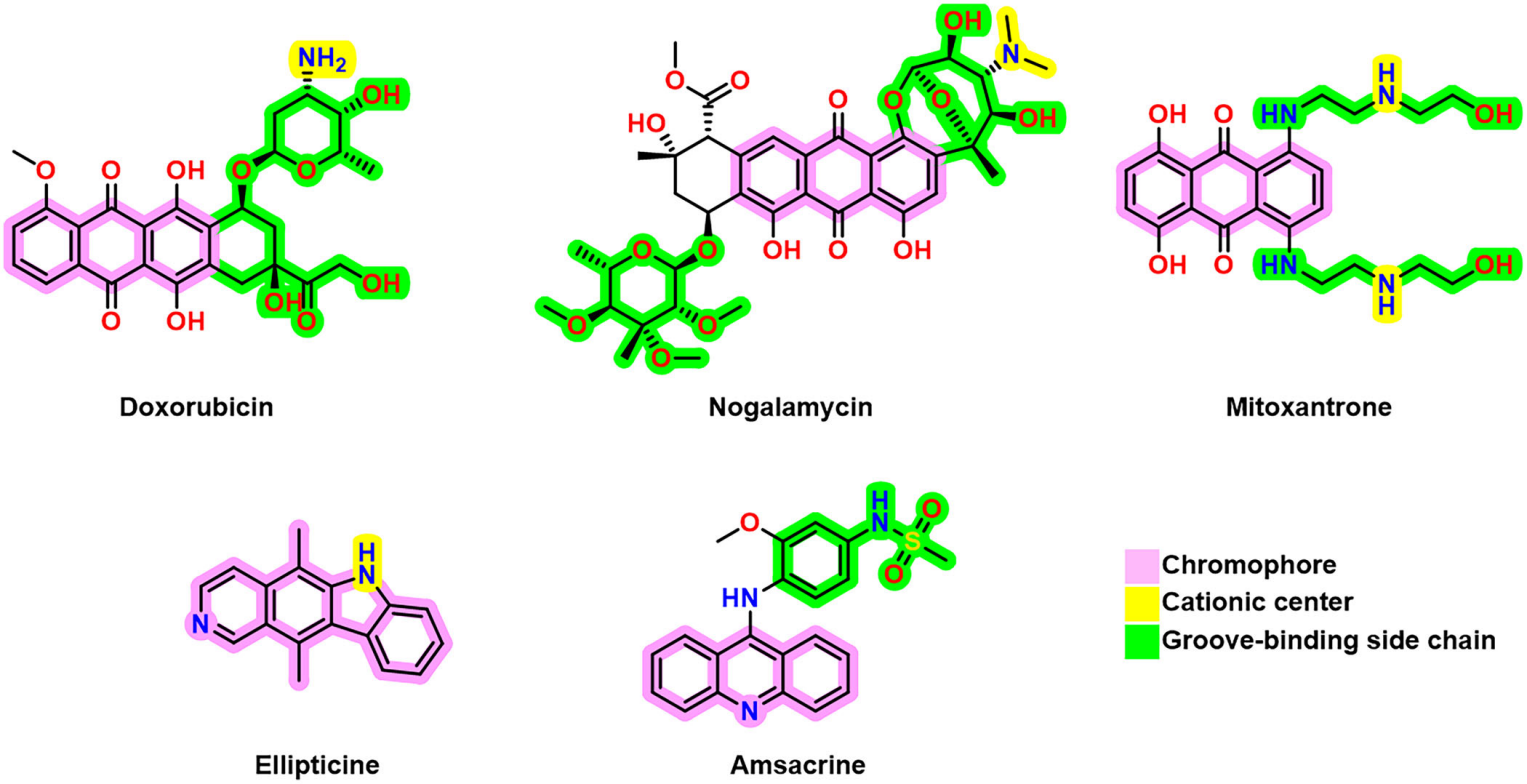
Some previously reported topoisomerase II inhibitors and DNA intercalators display their pharmacophoric characteristics.

DNA intercalators and TOP-2 poisons usually exhibit three pharmacophoric features; a polyaromatic planar structure (chromophore) to be placed between two neighbouring DNA nucleobases^14^, a cationic centre that interacts with the negatively charged phosphate group on DNA nucleobases, and a groove-binding site which stays in the DNA minor groove^15^. Some reported DNA intercalators and TOP-2 poisons are depicted in Figure 1^16–18^.

Immense research is taking place on the development of novel TOP-2 inhibitors. The journey for the development of a new drug candidate from drug discovery to FDA approval is an economically exhausting process with a low degree of success and uncertainty^19^^,^^20^. Repurposing drug candidates that are already approved by the FDA can surpass such time and cost resource consumption; meanwhile, reducing the incidence of developing a new agent with significant side effects that may impact the health of patients^21^^,^^22^. Repurposing FDA-approved drugs may also reduce the chemical consumption that is exerted in the development process which is a global quest for climate change. In addition, the utilisation of some *in-silico* approaches, such as molecular docking and molecular dynamics have a crucial role in the drug repurposing process by affording deep insights about the affinity of the repurposed compounds to the new specified targets and putting eyes on the possible capability of these compounds to be dedicated for new therapeutic uses along with their primary therapeutic uses^23–25^.

In this respect, antibiotics are fruitful candidates for drug repurposing. Numerous antibiotics may have protein and nucleic acid targets that exhibit similarities with those of the eukaryotic cells. For example; antibiotics that involve complexing with DNA or the associated proteins such as anthracycline and ionophores. This interaction with biologically relevant macromolecules provokes an antitumor potential of antibiotics. Those classes are either clinically approved or investigated for their antitumor activity^26–30^. In addition to providing a new therapeutic candidate, the repurposing of antibiotics may find a new role for drug molecules that lost their clinical relevance due to the development of bacterial resistance^31^.

Consequently, the rationale of this study is based on screening a reasonable number of available FDA-approved antibiotics that may interact with nucleic acid synthesis or replication. Also, they may share the same previously described pharmacophores commonly retained by most TOP-2 inhibitors. Therefore, 138 antibiotics belonging to aminoglycosides, lincosamides, macrolides, oxazolidinones, peptidyl transferases, streptogramins, sulphonamides, tetracyclines, quinolones, and other classes were *in silico* tested for their activity against TOP-2 using molecular docking. Then, some of the most promising and commercially available antibiotics were subjected to molecular dynamics simulations for 200 ns to get deep insights regarding their exact stability within the receptor pocket of Top-2. In addition, the MM-GBSA calculations for the frontier members were calculated. Additionally, the antitumor activities of the most promising candidates were investigated against three cancer cell lines (breast, liver, and colorectal) using DOX as a reference drug. Finally, the TOP-2 enzyme inhibition assay for the outstanding promising antibiotics compared to DOX as an FDA-approved TOP-2 inhibitor and DNA intercalator was carried out to confirm the proposed rationale.

## Materials and methods

**SP**, **RO**, **AZ**, **CL**, and **ER** (as ethyl succinate) were provided from NODQCAR at a purity > 99.87%. Human mammary tumours, hepatocellular carcinoma (HepG2), and colorectal cancer (HCT-116) cell lines were obtained from VACSERA; Egypt. RPMI 1640 doped with 10% FBS was provided from VACSERA; Egypt. The inhibitory potential against TOP-2 was assessed by the Topo GEN assay kit (TOPOGEN, CO, USA).

### *In silico* studies

#### Docking studies

The molecular docking studies were used to screen the binding interactions of the investigated antibiotics utilising the MOE 2019 suite^32^.

##### Preparation of the investigated compounds

PerkinElmer ChemOffice Suite 2017 toll was employed for drawing the screened compounds which were then made available for the docking process as previously described in the default procedure^33^^,^^34^. By importing them into one database and saving them as an MDB file, the screened compounds and the co-crystallised inhibitor (**EVP**) were ready for the docking process towards the human topoisomerase II-DNA complex.

##### Preparation of human topoisomerase II-DNA complex target

Using the protein data bank, the human topoisomerase II-DNA complex X-ray structure was downloaded (PDB code: 3QX3, https://www.rcsb.org/structure/3QX3). The target receptor was protonated, broken bonds were corrected, and the entire protein was energetically minimised to prepare for the molecular docking process as discussed previously in detail^35^^,^^36^.

##### Molecular docking against the prepared target complex

The general docking process was started after the file for the prepared active site was loaded. The docking process site was (Ligand atoms), the scoring methodology was (London dG) and the placement methodology was (triangle matcher). Rigid receptor docking was utilised as the refinement methodology and the scoring methodology was GBVI/WSA dG with the selection of the best 10 poses for each tested compound from 30 different poses. Subsequently, the investigated ligands’ MDB file was uploaded to the program and the calculations of the running docking were automatically executed. Following completion, the obtained poses were examined, and the best ones with the highest scores, acceptable RMSD values, and better ligand–enzyme interactions were chosen and saved for further investigation^37^^,^^38^.

#### Molecular dynamics simulations (MDs)

The MD simulations^39^^,^^40^ for the six studied complexes (**SP**-3QX3, **RO**-3QX3, **AZ**-3QX3, **CL**-3QX3, **ER**-3QX3, and **EVP**-3QX3) were performed using the Desmond package (Schrödinger LLC)^41^. Moreover, the thermal_mmgbsa.py python script of Schrodinger was applied to calculate the Molecular Mechanics Generalised Born Surface Area (MM-GBSA) energies^42^ for the aforementioned six complexes. The detailed methodologies were depicted in the Supplementary Information (SI 1 and SI 2).

### Biological evaluations

#### The antiproliferative activities of the most promising antibiotics against human breast, liver, and colorectal cancer cell lines

To determine the cytotoxic concentration of the tested antibiotics. Antibiotics were transferred into flat-bottomed cell culture microtiter plates (96 well plates, TPP-Swiss) at a concentration of 179.2, 133.5, 241.9, 227.3, and 205.5 µM for **SP**, **RO**, **AZ**, **CL**, and **ER**, respectively. An eight-stage four-fold dilution of the antibiotic with the culture media was performed. Cells were seeded (2 × 10^5^ cell/ml; 50 µL) in the wells and incubated for 24 h at 37 °C in a humidified and 5% CO_2_ atmosphere.

The viability of cells was determined using the tetrazolium-salt-based biochemical reaction, MTT assay^43^, where the cell’s treatment medium was decanted and washed thoroughly using a 200 µL buffering system (PBS) twice for 3 min each. This was followed by the addition of MTT (50 µL), and cells were incubated with the dye for another 4 h at 37 °C. The dye was discarded, and acidified isopropanol (0.4%, 50 µL) was dispensed to each well to dissolve the formazan crystals. The purple formazan colour was measured at a wavelength of 570 nm and a reference wavelength of 630 nm using an EL-x 800 microplate reader. Results were compared to a positive control (cells and solvent). DOX was used as standard (920 µM). Results were expressed as the concentration of antibiotics that inhibited the growth of cells by using the following equation: Viability % = OD test materials x 100/OD of cell control, then, the IC_50_ was calculated.

#### Topoisomerase II inhibitory assay

The drugs which showed good activity *in silico* were tested *in vitro* to estimate their inhibitory potential against TOP-2 using the TopGEN plasmid-based kit according to the user manual protocol TG1001^10^. Results were reported as IC_50_ and compared to DOX.

## Results and discussion

### *In silico* findings

#### Molecular docking studies

The molecular docking studies were used to screen the binding interactions of the investigated antibiotics utilising the MOE 2019 suite^32^^,^^42^^,^^44^. Molecular docking studies were employed to unveil the suggested binding interactions^45^^,^^46^ of these investigated compounds against the human topoisomerase II-DNA complex (PDB ID: 3QX3), with the native co-crystallised ligand (**EVP**) as a reference control.

**Figure 2.**
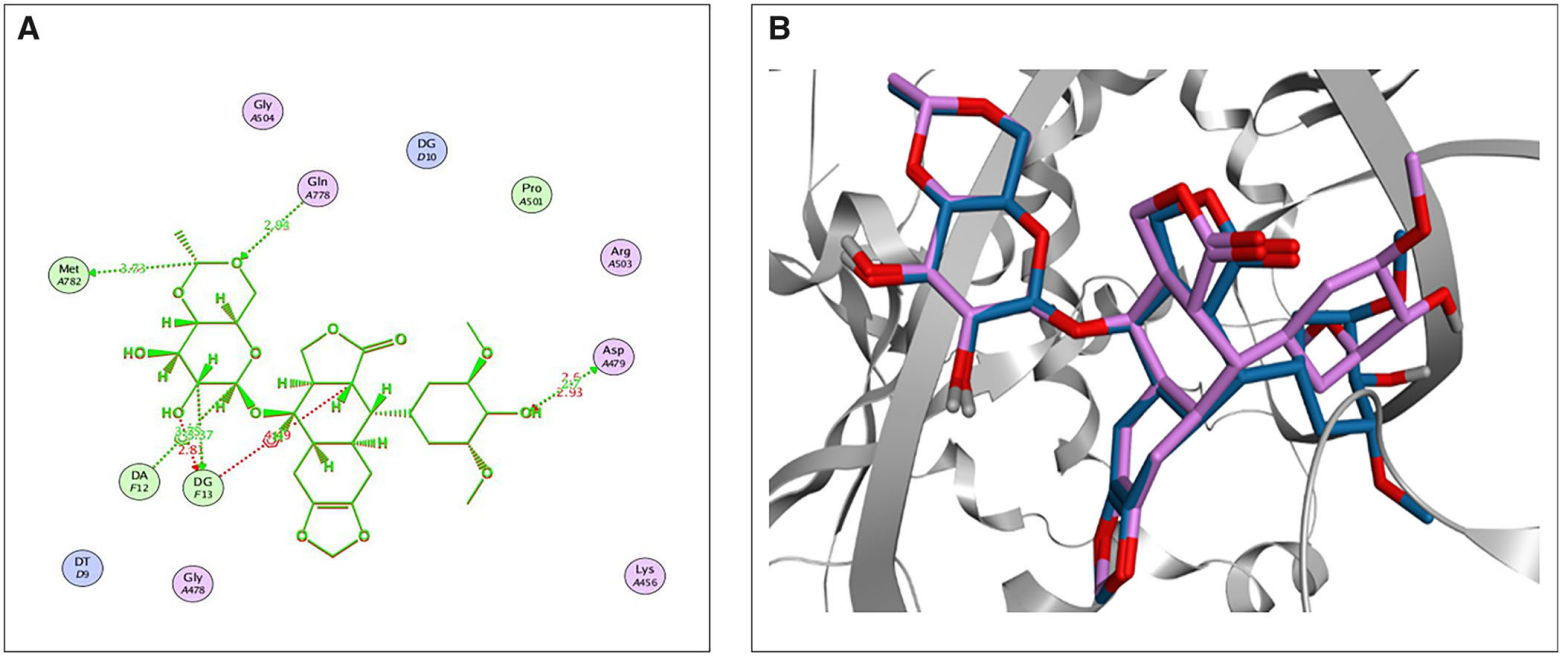
The 2D (A) and 3D (B) illustrations display the superimposition of the native co-crystallised **EVP** and redocked one (Mauve and Light Blue, respectively, in 3D picture) at the topoisomerase II-DNA complex (PDB entry**:** 3QX3) with RMSD value 1.42 Å.

First, the accuracy and suitability of the used force field and the docking protocols were validated via re-docking the co-crystallised **EVP** inhibitor against the human topoisomerase II-DNA complex target receptor. Consequently, a low RMSD value (1.42 Å) was obtained, enclosing the validity of the Amber10: EHT force field^20^^,^^47–49^; also, a similar binding mode was observed, as depicted in Figure 2. The 2D superimposition of the native and re-docked co-crystallised **EVP** is shown in Figure 2.

Molecular docking was conducted for screening the investigated compounds utilising the co-crystallised ligand (**EVP**) as a reference standard. Hence, deep insights into the binding interactions of the screened compounds against the DNA–topoisomerase II complex could be attained. The binding scores of the screened antibiotics towards the human topoisomerase II-DNA complex (PDB ID: 3QX3) compared to the co-crystallised inhibitor (**EVP**) are represented in the Supplementary Data (Table SI 1).

The binding scores, RMSD values, as well as the details of the binding interactions with their corresponding bond types for the most promising and commercially available five compounds (spiramycin (**SP**), roxithromycin (**RO**), azithromycin (**AZ**), clarithromycin (**CL**), and erythromycin (**ER**)), besides, the co-crystallised ligand (**EVP**), are depicted in Table 1.

**Table 1.** The binding free energy scores, RMSD values, and binding interactions of the most promising compounds (**SP**, **RO**, **AZ**, **CL**, and **ER**) along with the co-crystallised ligand (**EVP**) into DNA–topoisomerase II complex.

Compound	Score (kcal/mol)	RMSD (Å)	Interactions	Distance (Å)
**SP**	−12.07	1.68	ASP479/H-donor DG13/H-acceptor	2.71 3.03
**RO**	−11.97	1.57	ASP479/H-donor ARG503/H-acceptor LYS505/H-acceptor DT15/H-acceptor	2.87 3.32 3.11 2.99
**AZ**	−11.72	2.11	ASP479/H-donor GLU477/H-donor DT9/H-donor ARG503/H-acceptor	2.93 3.07 2.95 3.29
**CL**	−10.99	1.77	ASP479/H-donor LYS505/H-acceptor DG13/H-pi DG13/H-pi	2.86 3.31 3.87 4.25
**ER**	−10.83	2.09	DG10/H-donor ASP479/H-acceptor DG13/H-pi	3.01 3.33 4.57
**EVP**	−10.51	0.93	ASP479/H-donor MET782/H-donor DG13/H-donor GLN778/H-acceptor DA12/H-pi	2.70 3.72 3.37 2.94 3.75

It is worth noting that the nucleobases; Ade12, Gua13, Cyt8, Cyt11, and Thy9, and amino acids; ASP479, ARG503, GLN778, and MET782 could be regarded as the key binding sites of DNA–topoisomerase II complex^50^.

**Figure 3.**
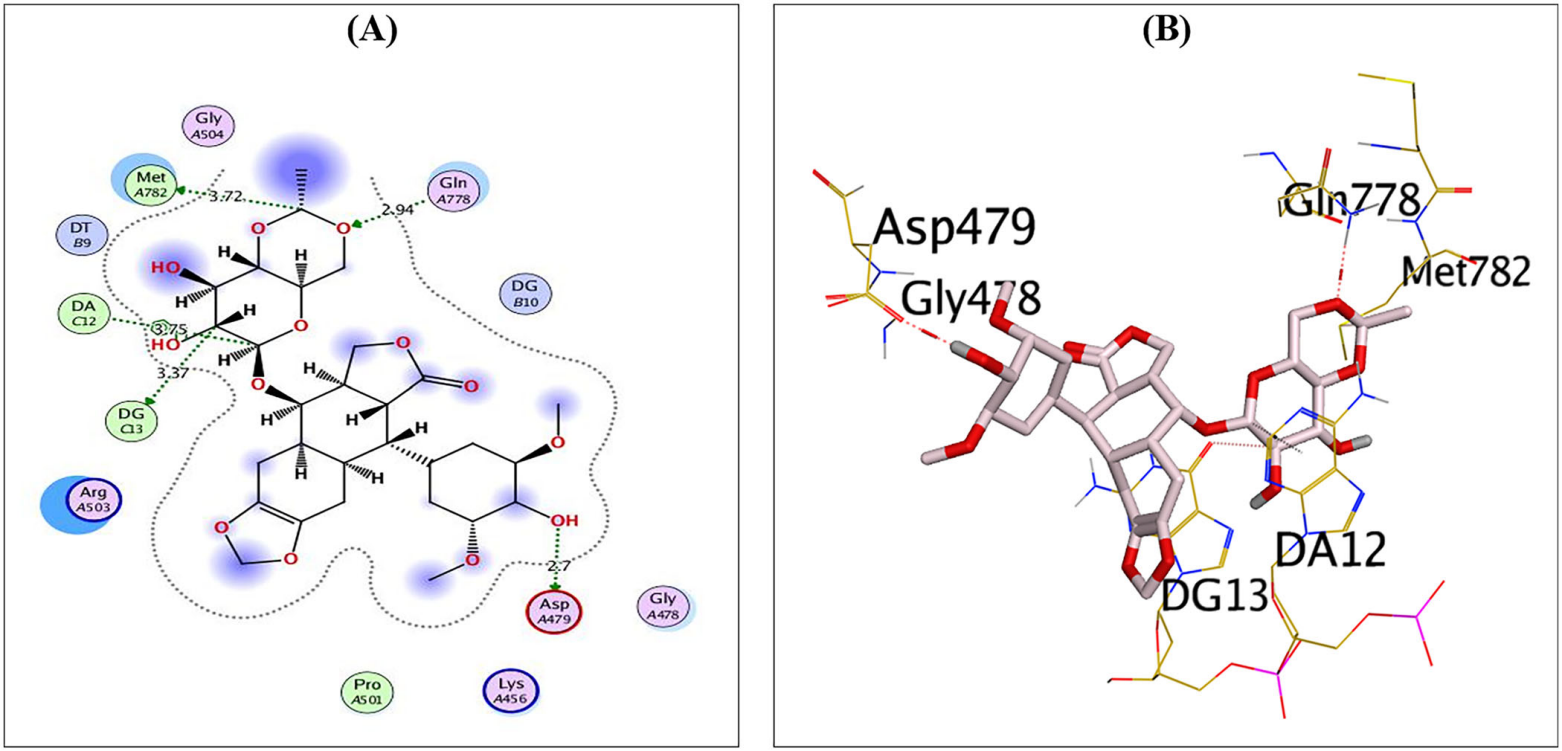
2D (A) and 3D (B) illustrations revealing the binding interactions of the re-docked co-crystallised ligand (**EVP**) at the topoisomerase II-DNA complex active site, where red dashed lines stand for H-bonds, and black ones stand for H-pi bonds.

Analysing the binding interactions of the re-docked native inhibitor (**EVP**) revealed that **EVP** displayed a binding score of −10.51 kcal/mol with an RMSD value of 0.93 Å. Furthermore, it is worth noting that the dioxane nucleus of **EVP** could form H-bonds with the key amino acids GLN778 and MET782 at distances of 2.94 and 3.72 Å, respectively. Besides, the pyran nucleus of **EVP** could interact with key nucleobases forming an H-bond with DG13 and an H-pi bond with DA12 at distances of 3.37 and 3.75 Å, respectively. Finally, the terminal –OH of the cyclohexyl moiety of **EVP** could form H-bond with the key amino acid ASP479 at a distance of 2.70 Å, as shown in Figure 3.

Compounds **AZ, CL,** and **ER** showed a docking score of −11.72, −10.99, and −10.83 kcal/mol, respectively, which is higher than that attained by the co-crystallised inhibitor.

**Figure 4.**
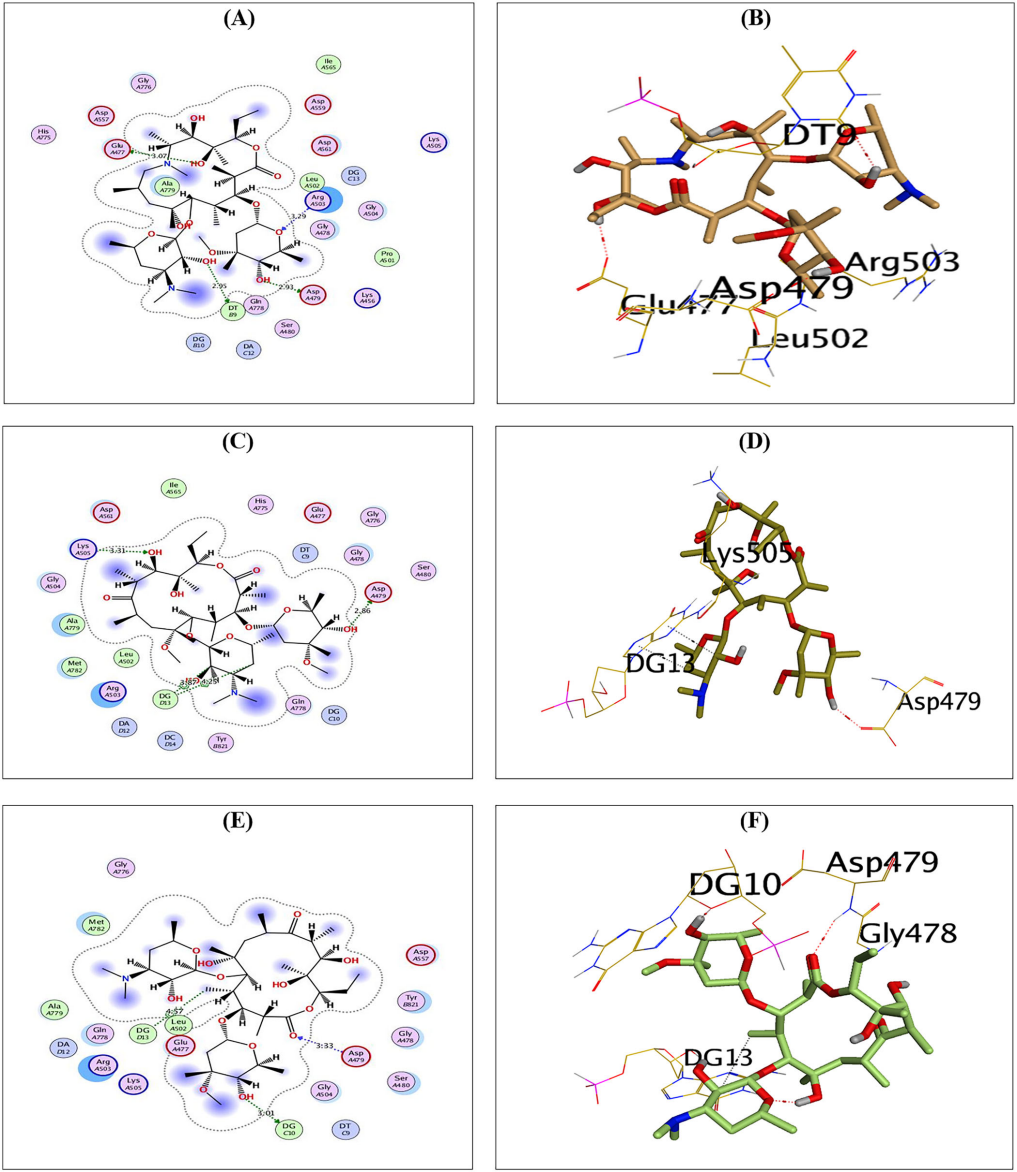
The 2D and 3D binding interactions of compounds **AZ** (A,B), **CL** (C,D), and **ER** (E,F) reveal their binding interactions at the topoisomerase II-DNA complex active site.

Compound **AZ** could compose H-bonds with the key amino acids ASP479, GLU477, and ARG503 at distances 2.93, 3.07, and 3.29 Å, respectively. Additionally, it could interact with the key nucleobase DT9 forming H-bond at a distance of 2.95 Å, as shown in Figure 4(A,B).

Compound **CL**, on the other hand, could compose H-bonds with the amino acids ASP479 and LYS505 at distances of 2.86 and 3.31 Å, respectively. In addition, it could interact with the key nucleobase DG13 forming two H-pi bonds at distances of 3.87 and 4.25 Å, as shown in Figure 4(C,D). Finally, compound **ER** could compose H-bond with the key amino acid ASP479 at a distance of 3.33 Å. Moreover, it could interact with the nucleobases DG10 and DG13 forming one H-bond (3.01 Å) and one H-pi bond (4.57 Å), as shown in Figure 4(E,F).

**Figure 5.**
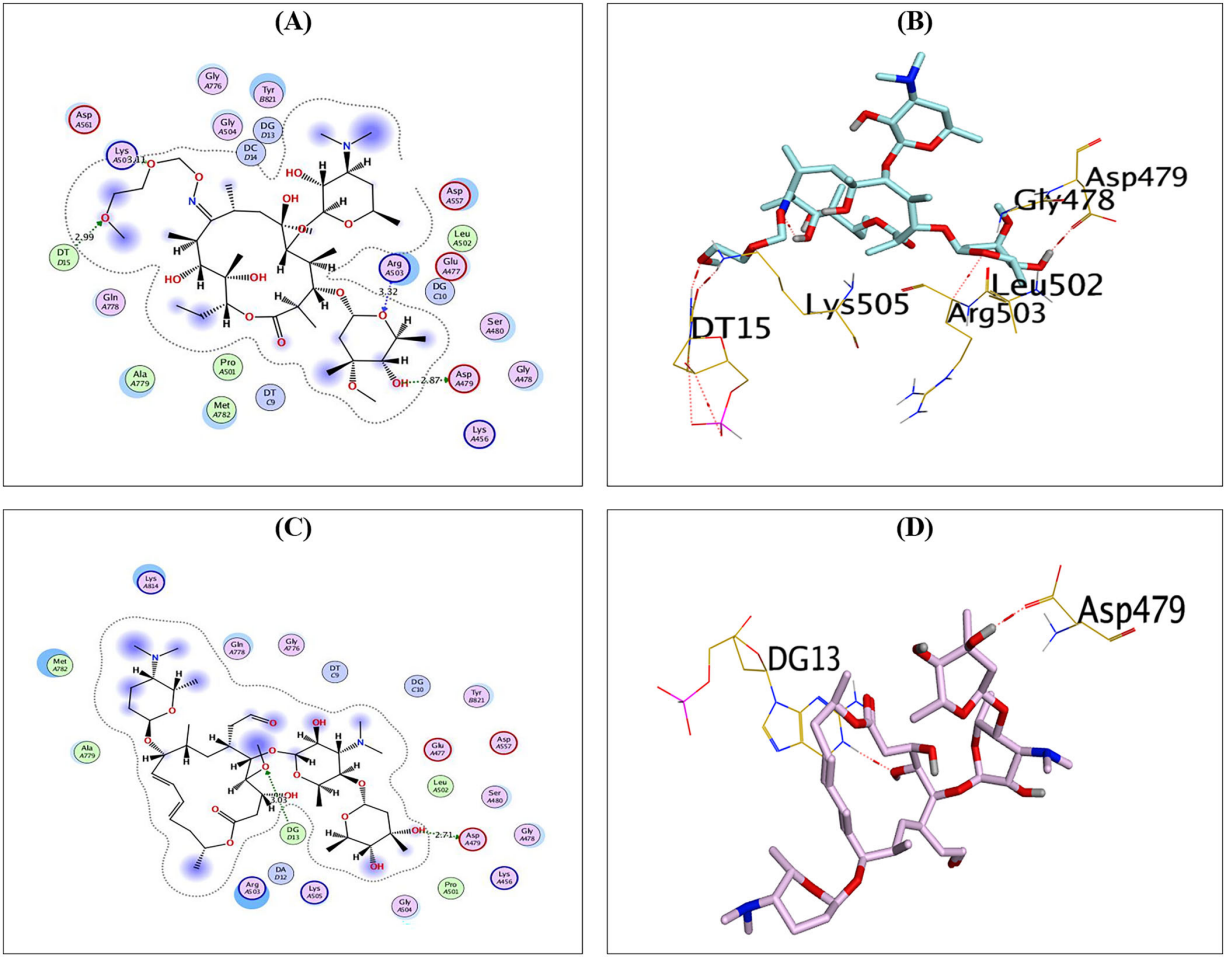
The 2D and 3D binding interactions of compounds **RO** (A,B), and **SP** (C,D) reveal their binding interactions at the topoisomerase II-DNA complex active site (PDB: 3QX3).

Furthermore, compounds **RO** and **SP** exhibited the best binding scores among selected compounds. Hence, compounds **RO** and **SP** revealed binding scores of −11.97 and −12.07 kcal/mol, which is superior to that of the co-crystallised inhibitor. Compound **RO** could compose H-bonds with the two key amino acids ASP479 and ARG503 and the amino acid LYS505 at distances 2.87, 3.32, and 3.11 Å, respectively. In addition, compound **RO** could interact with the nucleobase DT15 forming H-bond at a distance of 2.99 Å, as shown in Figure 5(A,B). On the other hand, compound **SP** could compose H-bonds with the key amino acid ASP479 at a distance of 2.71 Å and the key nucleobase DG13 at a distance of 3.03 Å, as shown in Figure 5(C,D).

#### Molecular dynamics simulations (MDs)

MD simulations were performed for 200 ns to monitor the exact behaviour of the superior and commercially available antibiotic members (**SP**, **RO**, **AZ**, **CL**, and **ER**) within the binding pocket of the topoisomerase II-DNA complex. This was done to clarify and deeply understand the protein–ligand interactions of the studied compounds^37^^,^^51^^,^^52^. Moreover, the **EVP** co-crystallised inhibitor was used as a reference.

##### RMSD analysis

In order to investigate the degree of deviation for the studied complexes regarding the initial position of their heavy atoms in a quantitative manner, the RMSD of the protein Cα atoms was recorded during the 200 ns of the simulation. It is an important parameter to judge the stability within the protein conformational changes all over the simulation time.

**Figure 6.**
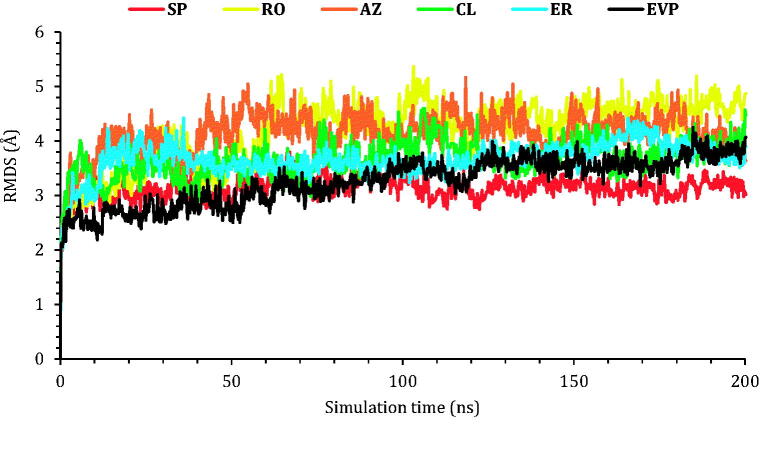
The RMSD of the six complexes (**SP**, **RO**, **AZ**, **CL**, and **ER**) within the topoisomerase II-DNA complex compared to the co-crystallised (**EVP**) inhibitor as a function of simulation time (200 ns).

The protein RMSD values of the six studied complexes are described in Figure 6. The protein RMSD for **SP** showed the most stable behaviour during the simulation time with an RMSD value of less than 3.00 Å. On the other hand, **EVP**, **CL**, and **ER** RMSD values were less than 4.00 Å indicating stable unchanged conformations for both proteins of **CL** and **ER** compared to that of the co-crystallised (**EVP**) inhibitor. Finally, the RMSD for **RO** and **AZ** proteins deviated at 60 and 40 ns, respectively, to 5.00 Å indicating slight conformational changes of the target TOP-2 protein.

**Figure 7.**
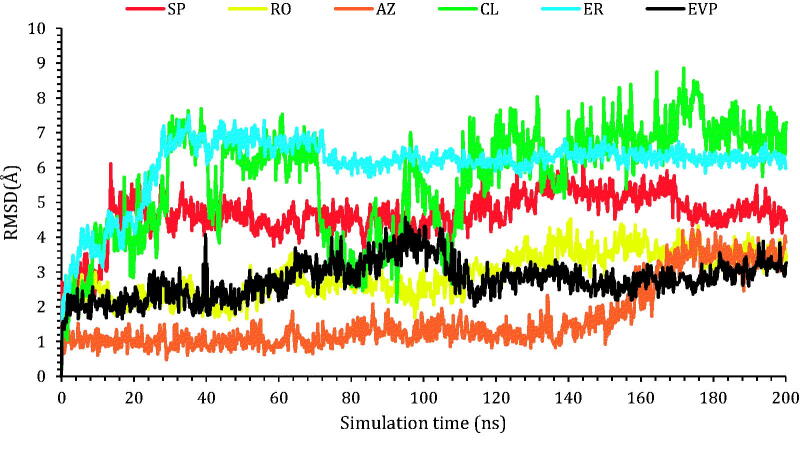
The RMSD of the ligands (**SP**, **RO**, **AZ**, **CL**, and **ER**) within the topoisomerase II-DNA complex compared to the co-crystallised (**EVP**) inhibitor as a function of simulation time (200 ns).

Next, the RMSD of the frontier ligands concerning their initial position inside the active site of the TOP-2 was reported in Figure 7. Among other compounds, **AZ** was the most stable inside the active site; it held its position with RMDS of 1.00 Å from the beginning of the simulation till around 150 ns, where it moved by 2.00 with respect to its recent position and held it towards the end of the simulation. The co-crystallised ligand (**EVP**) and compound **RO** showed similar behaviours. The RMSD of both compounds was around 3.00 Å concerning their original site. Compound **SP** moved by 5.00 Å concerning its initial position, while compound **ER** moved by 6.50 Å, where both stayed steady towards the end of the simulation. Finally, compound **CL** showed the highest RMSD, and could not hold a fixed position; the compounds moved by almost 7.00 Å concerning its initial position. Snapshots of the ligands–proteins during a different simulation time are presented in Figures SI 1–SI 6 (SI).

Figure SI 7 (SI) reports the RMSF (root mean square fluctuation). Where, most proteins fluctuated within the range of 3–4 Å, with *C*- and *N*- terminals fluctuating at around 8 Å. Such change is acceptable within such a large system.

##### Histograms analysis

**Figure 8.**
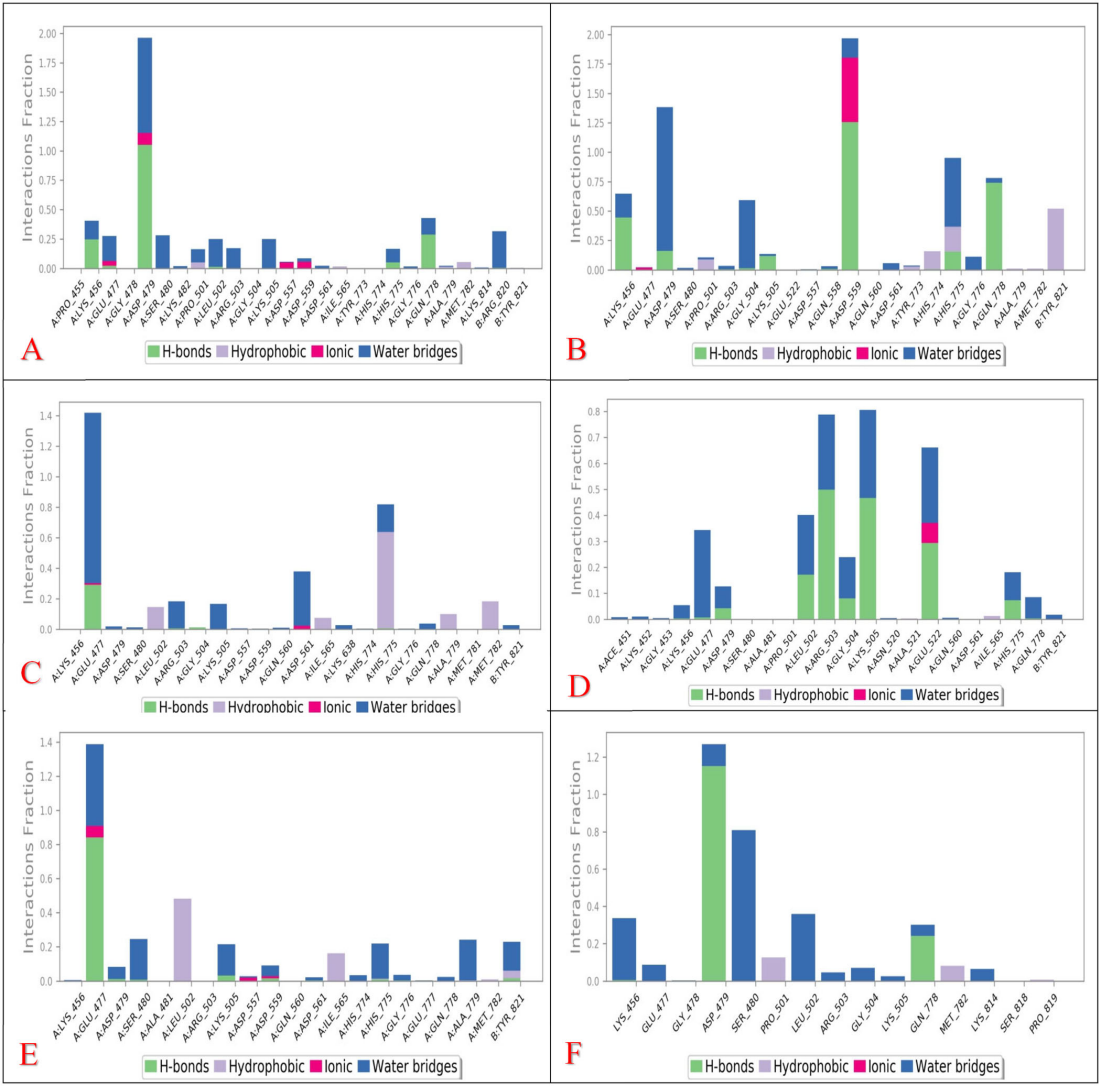
Histograms indicating the fractions of binding between the protein’s amino acids and its ligand for (A) **SP**-3QX3, (B) **RO**-3QX3, (C) **AZ**-3QX3, (D) **CL**-3QX3, (E) **ER**-3QX3, and (F) **EVP**-3QX3 complexes.

The protein–ligand interaction fractions for the six studied complexes (**SP**-3QX3, **RO**-3QX3, **AZ**-3QX3, **CL**-3QX3, **ER**-3QX3, and **EVP**-3QX3) are described in Figure 8 as histogram presentations.

The histogram of the **SP**-3QX3 complex (Figure 8(A)) showed that ASP479 contributed 195% of the interactions as the main amino acid. The interaction types were H-bonds (105%), water bridges (80%), and ionic interactions (10%).

However, the **RO**-3QX3 histogram (Figure 8(B)) showed that ASP559 was the principal amino acid in the interactions with **RO** with 195% as follows (H-bonds (125%), water bridges (60%), and ionic interactions (10%)). Also, ASP479 contributed 140% in the form of water bridges (120%) and H-bonds (20%).

On the other hand, the **AZ**-3QX3 histogram (Figure 8(C)) represented that GLU477 residue interaction reached 140% as water bridges (105%), H-bonds (30%), and ionic bonds (5%). Besides, HIS775 interactions were noticed to be 80% (hydrophobic (65%) and water bridges (15%)).

Moreover, the histogram of the **CL**-3QX3 complex (Figure 8(D)) represented that LYS505, ARG503, and GLU522 were the most bound amino acids to **CL** with 80%, 78%, and 66% interaction fractions, respectively. The type of interactions for LYS505 and ARG503 were H-bonds (48 and 50%) and water bridges (32 and 28%), respectively. Besides, GLU522 interactions were through H-bonds (30%), water bridges (28%), and ionic bonds (8%) as well.

Furthermore, the **ER**-3QX3 complex histogram (Figure 8(E)) clarified that GLU477 was the superior contributing amino acid to **ER** interactions with 140% through H-bonds (80%), water bridges (50%), and ionic bonds (10%).

Finally, regarding the histogram of the **EVP**-3QX3 complex (Figure 8(F)), ASP479 was noted to be the main amino acid included in the interactions with the co-crystallised **EVP** inhibitor (125%). It formed mainly H-bonds (115%) and a few water bridges (10%) to **EVP**. Also, the contributions of SER480 reached 80% through the formation of water bridges only to **EVP**.

Therefore, only **SP** (195%) and **RO** (140%) members showed a closely similar binding interaction to ASP479 amino acid of the binding pocket of the topoisomerase II-DNA complex compared to that of the co-crystallised **EVP** inhibitor (125%).

##### Heat maps analysis

The heat map analysis was considered to monitor the total number of interactions for each amino acid residue regarding the simulation time (200 ns). The heat maps for the six studied complexes (**SP**-3QX3, **RO**-3QX3, **AZ**-3QX3, **CL**-3QX3, **ER**-3QX3, and **EVP**-3QX3) are represented in Figures 9(A,B) and SI 8–SI 11, respectively.

Figure 9.**(**A) Heat map describing the protein–ligand interactions regarding the simulation time of 200 ns for **SP**-3QX3. (B) Heat map describing the protein–ligand interactions regarding the simulation time of 200 ns for **RO**-3QX3.

**Figure:**
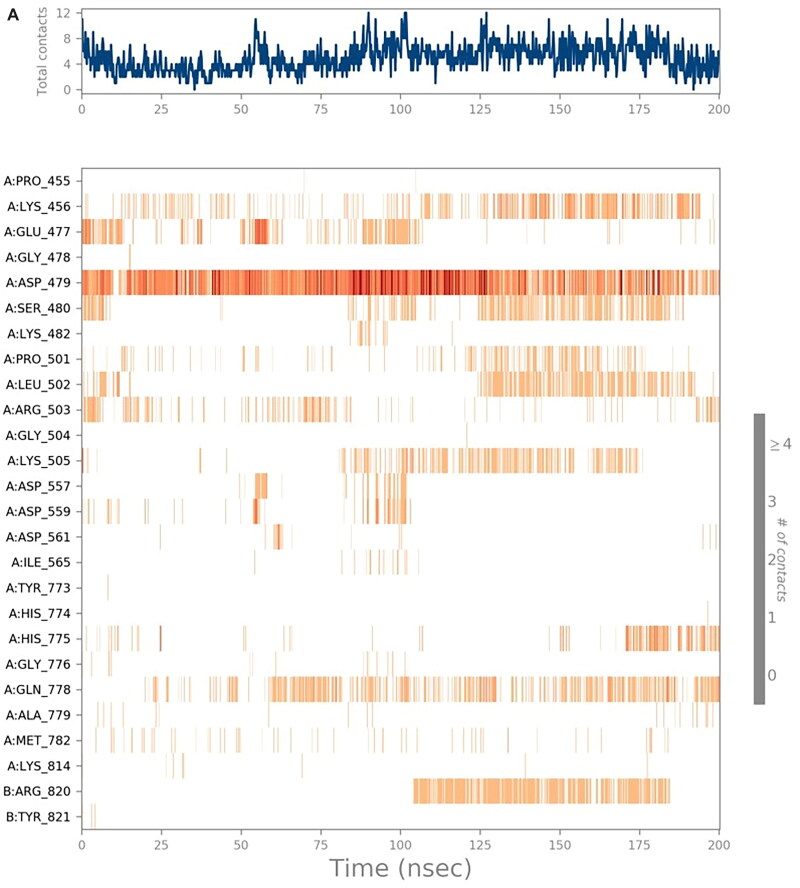

**Figure:**
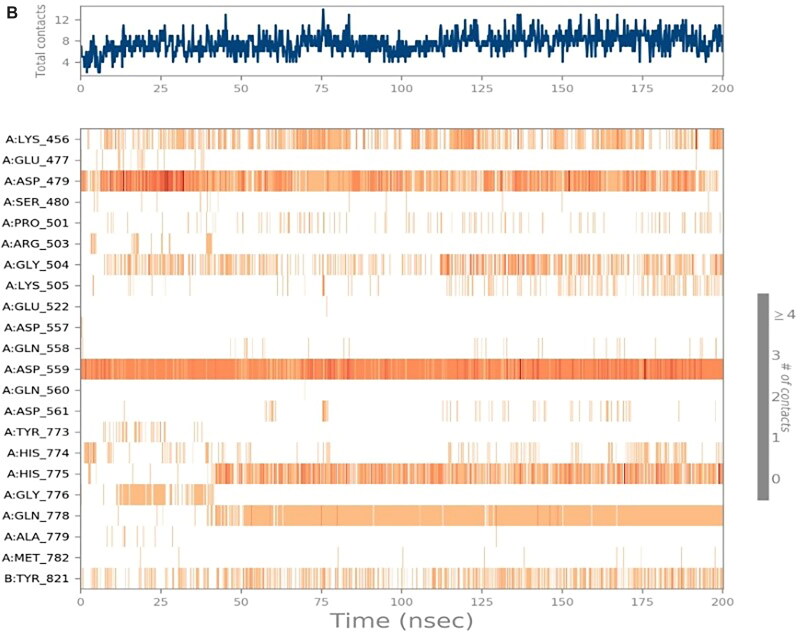

The heat map of the **SP**-3QX3 complex (Figure 9(A)) showed that ASP479 could form more than one type of interaction with **SP** all over the simulation time (200 ns). It showed the most apparent interactions from (0–10 and 20–130 ns) of the simulation time.

On the other hand, the **RO**-3QX3 heat map (Figure 9(B)) clarified that ASP559 interactions with **RO** were of high intensity all over the time of the simulation (200 ns). This was followed by Asp479 interactions, divided by the simulation time (200 ns), showing the most intense interactions from 10 to 30 ns.

Besides, **the AZ**-3QX3 heat map (Figure SI 8) showed that GLU447 amino acid interactions with **AZ** were more intense at (40–110 and 120–200 ns) of the simulation time. However, the HIS775 interactions were clearer from 50 to 190 ns of the simulation time.

However, the heat map of the **CL**-3QX3 complex (Figure SI 9) notified that LYS505 interactions were almost from 0–30 and 75–200 ns. ARG503 interactions on the other hand were distributed equally all over the simulation time (200 ns). Also, GLU522 interactions with **CL** were more explicit from 140 ns until the end of the simulation.

Moreover, the heat map of the **ER**-3QX3 complex (Figure SI 10) represented the equal distribution of GLU477 interactions to **ER** from the beginning to the end of the simulation time (200 ns).

Finally, the **EVP**-3QX3 heat map (Figure SI 11) clarified that the interactions of ASP479 to the co-crystallised **EVP** inhibitor were of high intensity from the start to the end of the simulation time (200 ns). Besides, SER480 contributions were mostly from 35 to 190 ns of the simulation time.

#### MM-GBSA calculations

The average MM-GBSA binding energy was calculated using the thermal_mmgbsa.py python script of Schrodinger^41^^,^^53^ to calculate Van der Waals, Hydrogen-bonding, Generalised Born electrostatic solvation, Coulomb, Lipophilic, and Covalent binding energies. The calculated energies for compounds **SP**, **RO**, **AZ**, **CL**, **ER**, and **EVP** at the topoisomerase II-DNA complex are represented in Table 2.

**Table 2.** Prime MM-GBSA energies for compounds **SP**, **RO**, **AZ**, **CL**, **ER**, and **EVP** at the binding pocket of the topoisomerase II-DNA complex.

Complex	**SP**-3QX3	**RO**-3QX3	**AZ**-3QX3	**CL**-3QX3	**ER**-3QX3	**EVP**-3QX3
ΔG Binding	−60.62	−63.90	−69.78	−48.76	−73.77	−72.45
Coulomb	−131.04	−95.20	−184.81	−79.61	−100.05	−25.78
Covalent	3.26	1.99	1.42	1.42	0.41	2.00
H-bond	−0.63	−2.08	−0.86	−1.31	−0.80	−1.26
Lipo	−11.08	−14.04	−15.16	−10.13	−14.47	−17.81
Solv_GB	145.96	113.13	199.54	93.02	115.32	37.77
VdW	−67.09	−67.70	−69.91	−52.15	−74.17	−67.37
St. dev.	28.57	31.01	5.96	4.82	4.67	3.40

Lipo: Lipophilic energy; Solv_GB: Generalised Born electrostatic solvation energy; VdW: Van der Waals energy; and St. dev.: standard deviation.

Based on the results in Table 2, we can observe that the ΔG Binding (−73.77 kcal/mol) and VdW (−74.17 kcal/mol) energies of **ER** were superior to other members including the co-crystallised **EVP** inhibitor. Also, the Coulomb (−184.81 kcal/mol) and Solv_GB (199.54 kcal/mol) energies for **AZ** were higher than those of the studied candidates. The Lipo energy of **AZ** (−15.16 kcal/mol) was very close to that of **EVP** (−17.81 kcal/mol). On the other hand, **SP** showed the highest Covalent energy (3.26 kcal/mol) within the binding pocket of the topoisomerase II-DNA complex. However, **RO** formed the best H-bond energy (−2.08 kcal/mol) towards the binding pocket of the topoisomerase II-DNA complex compared to the other examined drugs.

### Biological evaluations

#### The antiproliferative activities of the most promising antibiotics against human breast, liver, and colorectal cancer cell lines

**Figure 10.**
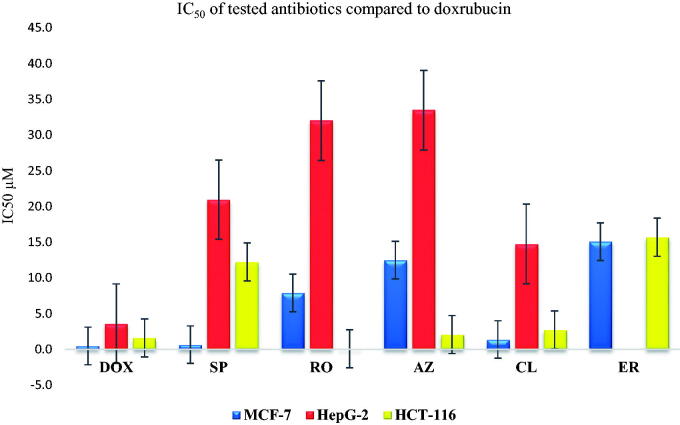
Inhibitory potential (IC_50_) of the most promising and commercially available antibiotics (**SP**, **RO**, **AZ**, **CL**, and **ER**) against MCF-7, HepG2, and HCT-116.

The cytotoxicity (IC_50_) assay was tested against three cancer cell lines namely breast cancer cell line (MCF-7), hepatocellular carcinoma (HepG-2), and human colorectal carcinoma (HCT-116) utilising the MTT assay. In addition, the IC_50_ was compared to that of DOX as a reference drug. Results showed a comparable IC_50_ to that attained by DOX (Figure 10).

**SP** showed a promising anticancer potential on the MCF-7 cell line (IC_50_ 0.67 ± 0.43 µM). This was followed by a weaker activity towards both HepG-2 (20.94 ± 1.33 µM) and HCT-116 (12.23 ± 3.27), respectively. On the other hand, **RO** exhibited an antiproliferative activity of 7.9 ± 0.79, 32 ± 0.18, and 0.097 ± 0.024 µM as IC_50_ on MCF-7, HepG-2, and HCT-116, respectively, representing moderate potential on MCF-7 and HCT-116, with a strong potential against HCT-116^5^,^2^^,^^55^. However, **AZ** exhibited a good anticancer potential against the HCT-116 cell line (2.08 ± 0.05 µM) compared to 1.6 ± 0.05 µM for DOX. Furthermore, moderate and weak anticancer activities were recorded for **AZ** with average IC_50_ of 7.9 ± 0.79 and 32 ± 0.18 µM for MCF7 and HepG-2, respectively, compared to 0.49 ± 0.1 (MCF7) and 3.6 ± 0.07 (HepG-2) µM for DOX. These data are supported by previous reports, indicating AZ’s close potential for Chronic Myeloid Leukaemia^56^. On the other hand, **CL** exhibited a promising antiproliferative pattern on different cell lines where 1.39 ± 0.15, 14.77 ± 0.15, and 2.74 ± 0.42 µM were recorded as the IC_50_ in MCF-7, HepG-2, and HCT-116 cell lines, respectively, representing good activity on MCF-7 and HCT-116, besides a moderate antiproliferative potential towards the HepG-2 type. These results are similar to previous reports, which highlight the potential of **CL** as an anticancer agent^54^^,^^57–60^. Moreover, **ER** showed weak to moderate inhibitory potential with IC_50_ of 15.07 ± 2.7 and 15.69 ± 1.32 µM against MCF-7 and HCT-116 cell lines, respectively^61^.

#### Topoisomerase II inhibitory activity

The potential of the tested drugs to inhibit the ability of TOP-2 to decatenate an intertangled mass of DNA was assessed. In addition, the selected macrolides were further investigated to determine their inhibitory potential on the TOP-2 enzyme using DOX (an anthracycline antibiotic with potent inhibition towards TOP-2) as a positive control. The acquired results agreed with the cytotoxicity assay, and the obtained data indicated that the tested antibiotics have a promising inhibitory potential against the TOP-2 enzyme relative to DOX.

**Figure 11.**
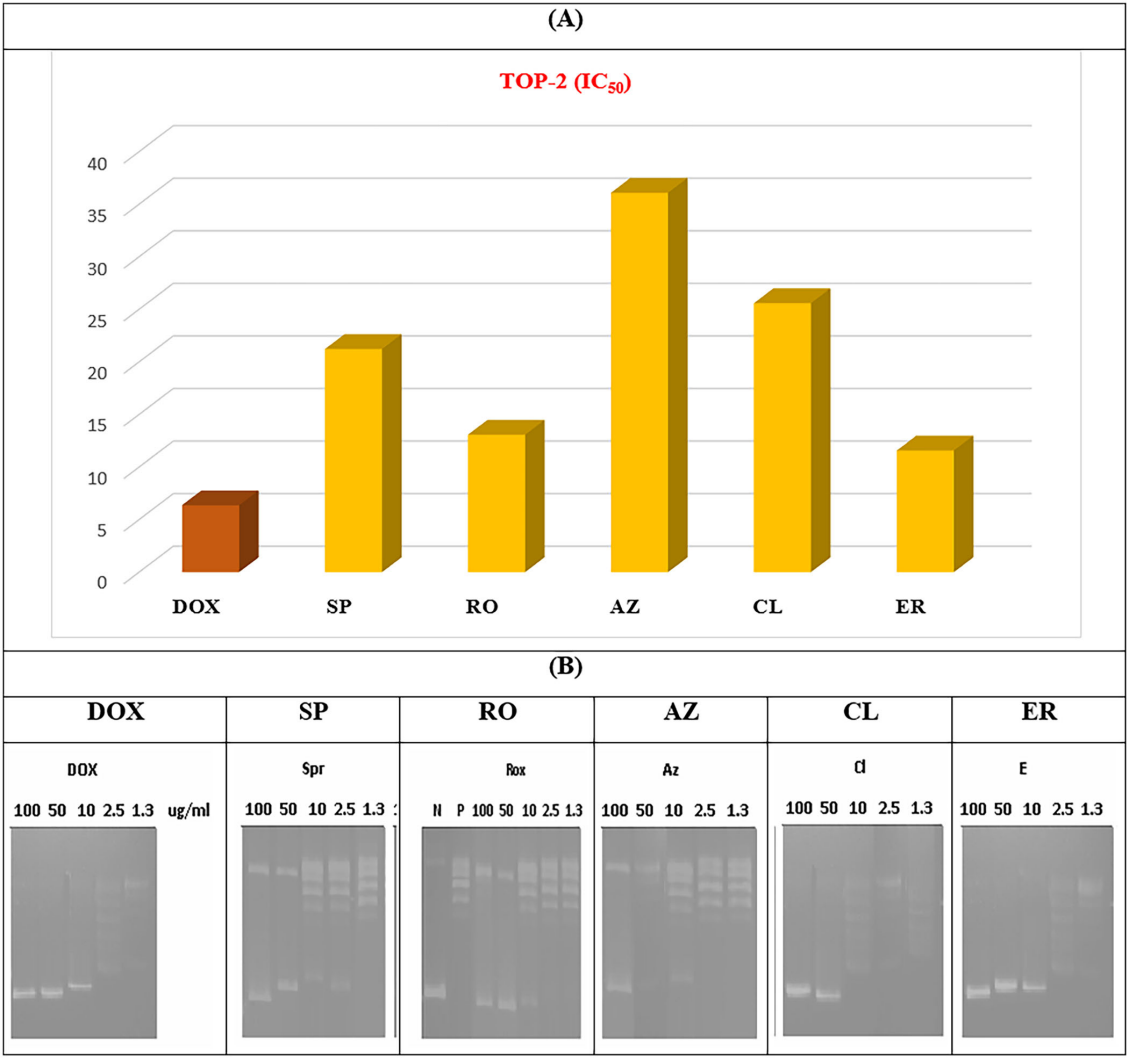
(A) The measured IC_50_ of tested antibiotics (**SP**, **RO**, **AZ**, **CL**, and **ER**) on TOP-2 compared to DOX. (B) Gel electrophoresis image representing the variable decatenation potential of DNA by TOP-2 as inhibited by DOX and the tested antibiotics (**SP**, **RO**, **AZ**, **CL**, and **ER**). The N Lane represents no TOP-2 activity while the P lane is the uninhibited enzyme.

The recorded IC_50_ for DOX (positive control) was 6.37 ± 0.38 µM. In addition, potent TOP-2 inhibitory activity was recorded for **ER** and **RO** with IC_50_ of 11.58 ± 0.70 and 13.07 ± 0.77 µM, respectively. On the other hand, **AZ** showed moderate activity with an IC_50_ of 36.09 ± 2.11 µM. Finally, **CL** and **SP** exhibited moderate IC_50_ of 25.58 ± 1.08 and 21.23 ± 1.19 µM, respectively (Figure 11).

The order of IC_50_ of different antibiotics was slightly different in cytotoxicity and enzyme assay, especially for **ER**. Variations may explain the tested antibiotics’ cellular/nuclear permeability in different cell lines.

### Structure-activity relationship (SAR)

**Figure 12.**
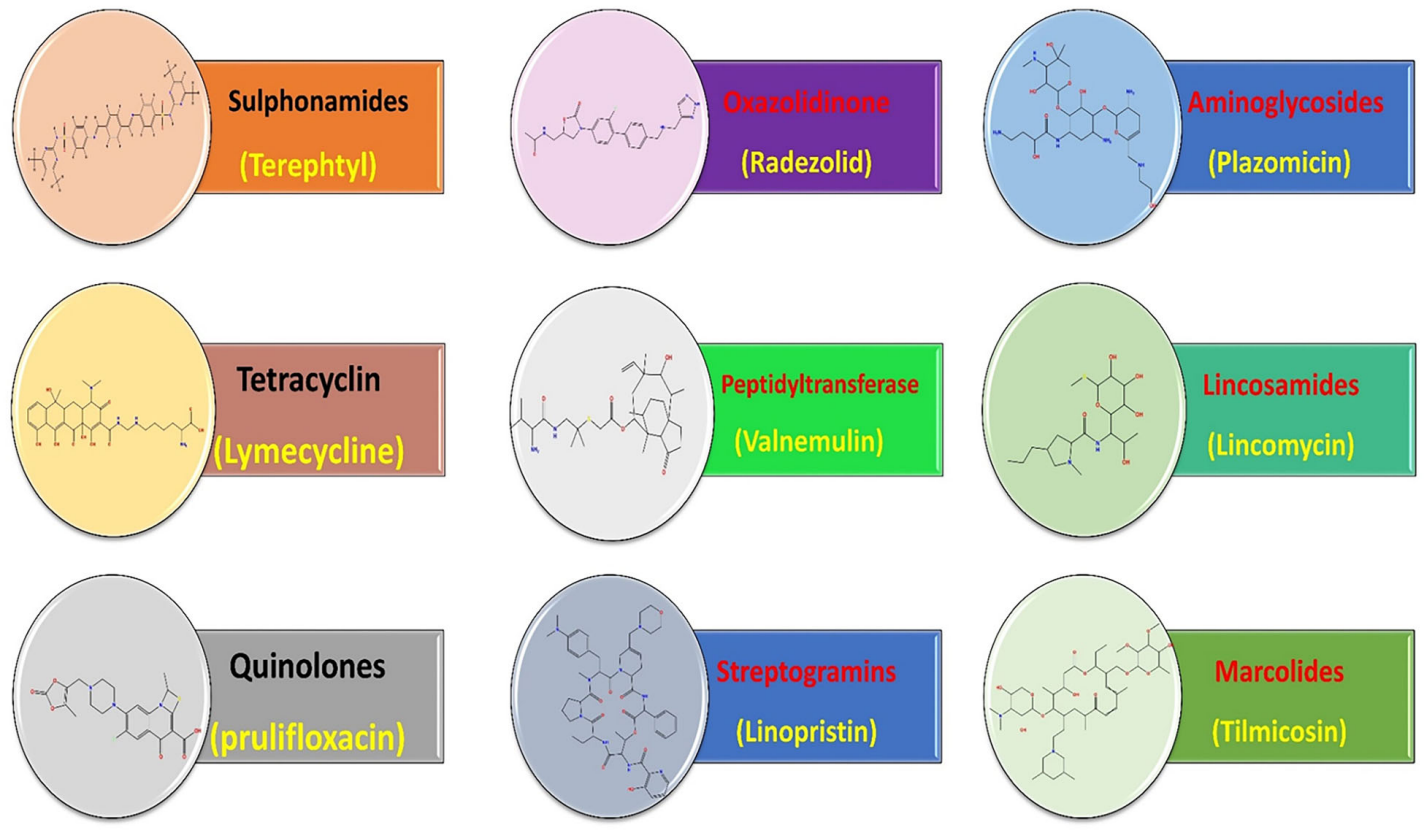
SAR study for the affinity of the examined 138 antibiotics as topoisomerase II inhibitors and DNA intercalators.

Based on the molecular docking binding scores for the tested antibiotics (Table SI 1), a SAR study was conducted opening eyes to promising anticancer pharmacophores encountered by the screened antibiotics. Interestingly, the following outcomes were attained (Figure 12):

- Among the screened antibiotics, it was disclosed that macrolides and streptogramins exhibited the most promising binding scores and, thus, higher anticancer potential.
- Moreover, among screened aminoglycosides, it was revealed that **plazomicin** displayed the best binding affinity to TOP-2, whereas, among screened lincosamides, it was unravelled that **lincomycin** showed the best binding score.
- Besides, regarding screened macrolides, it was shown that all compounds experienced promising binding scores that are better than that attained by the co-crystallised ligand assuring their stability at the target receptor. In particular, it was disclosed that **tilmicosin** showed the best binding score among the investigated macrolides.
- Furthermore, considering screened oxazolidinone, it was disclosed that **radezolid** showed the best binding affinity to the target receptor, whereas, among screened peptidyltransferase, it was revealed that **valnemulin** has the best docking score.
- In addition, regarding screened streptogramins, it was shown that **linopristin**, **pristinamycin IA**, **quinupristin**, and **virginiamycin S1** antibiotics exhibited promising docking scores that are better than that attained by the co-crystallised ligand. In particular, it was disclosed that **linopristin** showed the best docking score among the screened streptogramins.
- Additionally, regarding sulphonamides, tetracyclines, and quinolones, it was unveiled that the compounds with the best binding score in their corresponding class were **terephtyl**, **lymecycline**, and **prulifloxacin**, respectively.
- It is worth noting that, among screened antibiotics, sulphonamides and quinolones exhibited the lowest binding scores, and thus, less anticancer potential could be attained.

## Conclusions

This research aims to identify a potential TOP-2 inhibitor that is considered a promising molecular target for cancer therapy. Herein, the molecular docking studies of 139 antibiotics belonging to different classes revealed that **SP**, **RO**, **AZ**, **CL**, and **ER** were among the most promising and commercially available members. Afterward, the MD simulations for 200 ns clarified that **AZ** was the most stable inside the active site of the topoisomerase II-DNA complex, followed by the co-crystallised ligand (**EVP**) and **RO,** which showed similar behaviours according to the RMSD analysis. Also, the histogram analysis showed that only **SP** and **RO** members showed a closely similar binding interaction to ASP479 crucial amino acid of the TOP-2 receptor, compared to that of the co-crystallised **EVP** inhibitor. Moreover, based on the MM-GBSA calculations, it was observed that the ΔG Binding energy of **ER** (-73.77 kcal/mol) was superior to the other MD-studied compounds, including the co-crystallised **EVP** inhibitor. On the other hand, the MTT assay against MCF-7, HepG-2, and HCT-116 cancer cell lines clarified that **SP** and **CL** showed promising anticancer potentials on the MCF-7 cell line with IC_50_ values of 0.67 ± 0.43 and 1.39 ± 0.15 µM, respectively, compared to that of DOX (0.49 ± 0.1 µM). Besides, **AZ** and **CL** exhibited good anticancer potentials against the HCT-116 cell line (2.08 ± 0.05 and 2.74 ± 0.42 µM, respectively) compared to 1.6 ± 0.05 µM for DOX. Furthermore, the TOP-2 enzyme inhibition assay was carried out to confirm the proposed rationale compared to DOX as a TOP-2 inhibitor and DNA intercalator. Notably, potent TOP-2 inhibitory potentials were recorded for **ER** and **RO** with IC_50_ values of 11.58 ± 0.51 and 13.07 ± 0.65 µM, respectively, compared to DOX (6.37 ± 0.21 µM). Additionally, the SAR study revealed that macrolides and streptogramins exhibited the most promising binding scores among the tested antibiotics and thus expected better anticancer potentials. Finally, it is recommended to evaluate all the superior antibiotics (either examined or not) using more advanced preclinical and clinical studies to get a more accurate conclusion regarding their antitumor activities.

## Supplementary Material

Supplemental Material
