# Supplementary material for: Investigating the potential anticancer activities of antibiotics as topoisomerase II inhibitors and DNA intercalators: in vitro, molecular docking, molecular dynamics, and SAR studies
Source: J Enzyme Inhib Med Chem. 2023 Jan 26;38(1):2171029. doi: 10.1080/14756366.2023.2171029 (PMC9881673; doi:10.1080/14756366.2023.2171029)
Supplement: Supplemental Material [file IENZ_A_2171029_SM2761.pdf]

Investigating the Potential Anticancer Activities of Antibiotics as Topoisomerase II Inhibitors and DNA Intercalators: *In Vitro*, Molecular Docking, Molecular Dynamics, and SAR Studies

Faten Farouk[1], Ayman Abo Elmaaty[2], Ahmed Elkamhawy[3,4], Haytham O. Tawfik[5], Radwan Alnajjar[6,7,8], Mohammed A. S. Abourehab[9], Mohamed A. Saleh[10,11], Wagdy M. Eldehna[12,13], and Ahmed A. Al-Karmalawy[1,*]

[1] Pharmaceutical Chemistry Department, Faculty of Pharmacy, Ahram Canadian University, 6th of October City, Giza 12566, Egypt.

[2] Department of Medicinal Chemistry, Faculty of Pharmacy, Port Said University, Port Said 42526, Egypt.

[3] BK21 FOUR Team and Integrated Research Institute for Drug Development, College of Pharmacy, Dongguk University-Seoul, Goyang, 10326, Republic of Korea.

[4] Department of Pharmaceutical Organic Chemistry, Faculty of Pharmacy, Mansoura University, Mansoura, 35516, Egypt.

[5] Department of Pharmaceutical Chemistry, Faculty of Pharmacy, Tanta University, El Giesh street, Tanta, 31527, Egypt.

[6] Department of Chemistry, Faculty of Science, University of Benghazi, Benghazi, Libya.

[7] PharmD, Faculty of Pharmacy, Libyan International Medical University, Benghazi, Libya.

[8] Department of Chemistry, University of Cape Town, Rondebosch 7701, South Africa.

[9] Department of Pharmaceutics, Faculty of Pharmacy, Umm Al-Qura University, Makkah 21955, Saudi Arabia.

[10] Department of Clinical Sciences, College of Medicine, University of Sharjah, Sharjah 27272, the United Arab Emirates.

[11] Department of Pharmacology and Toxicology, Faculty of Pharmacy, Mansoura University, Mansoura 35516, Egypt.

[12] Department of Pharmaceutical Chemistry, Faculty of Pharmacy, Kafrelsheikh University, Kafrelsheikh 33516, Egypt.

[13] School of Biotechnology, Badr University in Cairo, Badr City 11829, Egypt.

* Corresponding author:

Ahmed A. Al-Karmalawy: Email: akarmalawy@acu.edu.eg

ORCID: 0000-0002-8173-6073

Supplementary Material

Table SI 1: The binding scores of the screened antibiotics (**1-138**) toward the human topoisomerase II-DNA complex (PDB ID: 3QX3) compared to the co-crystallized inhibitor (**EVP**).

No.	Antibiotic	Score	RMSD
**Aminoglycosides**			
**1**	**Amikacin**	-9.09	2.01
**2**	**Apramycin**	-8.70	1.84
**3**	**Arbekacin**	-8.66	1.20
**4**	**Astromicin**	-7.61	1.21
**5**	**Bekanamycin**	-8.19	1.87
**6**	**Butirosin**	-9.40	1.43
**7**	**Clomocycline**	-7.84	1.94
**8**	**Demeclocycline**	-7.43	1.05
**9**	**Dibekacin**	-8.25	1.18
**10**	**Gentamicin**	-8.35	2.14
**11**	**Hygromycin b**	-8.26	1.83
**12**	**Isepamicin**	-8.83	2.03
**13**	**Kanamycin**	-8.03	1.80
**14**	**Micronomicin**	-8.07	1.83
**15**	**Neomycin**	-9.14	2.00
**16**	**Netilmicin**	-8.59	2.16
**17**	**Nourseothricin**	-8.35	1.97
**18**	**Paromycin**	-9.55	1.90
**19**	**Plazomicin**	-9.57	1.95
**20**	**Ribostamycin**	-8.08	1.43
**21**	**Sisomicin**	-8.24	1.46
**22**	**Spectinomycin**	-6.45	0.51
**23**	**Streptomycin**	-8.81	1.99
**24**	**Tobramycin**	-8.31	1.50
**25**	**Verdamicin**	-8.27	2.19
**Lincosamides**			
**26**	**Clindamicin**	-7.59	1.65
**27**	**Lincomycin**	-8.00	1.49
**28**	**Pirlimycin**	-7.63	2.17
**Marcolides**			
**29**	**Azithromycin**	-11.72	2.11
**30**	**Clarithromycin**	-10.99	1.77
**31**	**Erythromycin**	-10.83	2.09
**32**	**Telirithromycin**	-10.94	2.29

**33**	**Boromycin**	-12.20	1.48
**34**	**Carbomycin**	-11.26	2.29
**35**	**Josamycin**	-11.93	1.90
**36**	**Kitasamycin**	-11.91	2.00
**37**	**Midecamycin**	-11.57	1.79
**38**	**Miocamycin**	-12.01	1.46
**39**	**Oleandomycin**	-10.42	2.23
**40**	**Rokitamycin**	-12.03	1.70
**41**	**Roxithromycin**	-11.97	1.57
**42**	**Spiramycin**	-12.07	1.68
**43**	**Tilmicosin**	-12.77	2.20
**44**	**Troleandomycin**	-11.89	1.76
**45**	**Tulathromycin**	-11.53	2.12
**46**	**Tylosin**	-11.61	2.14
**Oxazolidinone**			
**47**	**Eperezolid**	-7.79	1.18
**48**	**Linezolid**	-6.70	1.60
**49**	**Posizolid**	-8.26	1.23
**50**	**Radezolid**	-8.47	1.71
**51**	**Ranbezolid**	-8.15	1.62
**52**	**Sutezolid**	-7.00	0.60
**53**	**Tedizolid**	-7.41	0.61
**Peptidyltransferase**			
**54**	**Azamulin**	-7.83	1.51
**55**	**Azidamfenicol**	-6.16	1.76
**56**	**Chloramphenicol**	-6.29	1.97
**57**	**Florfenicol**	-6.36	1.92
**58**	**Lefamulin**	-8.38	1.52
**59**	**Retapamulin**	-8.36	1.70
**60**	**Thiamphenicol**	-6.29	0.92
**61**	**Tiamulin**	-8.34	1.63
**62**	**Valnemulin**	-9.3	1.97
**Streptogramins**			
**63**	**Dalfopristin**	-9.94	2.06
**64**	**Flopristin**	-8.69	1.11
**65**	**Pristinamycin IIa**	-8.81	2.10
**66**	**Linopristin**	-13.54	1.94
**67**	**Pristinamycin IA**	-11.32	1.84
**68**	**Quinupristin**	-13.26	2.17
**69**	**Virginiamycin S1**	-11.52	1.64
**Sulphonamides**			

**70**	**Sulfacetamide**	-5.42	1.25
**71**	**Sulfadiazine**	-5.47	1.41
**72**	**Sulfadimethoxine**	-6.42	0.89
**73**	**Sulfadoxine**	-6.56	1.41
**74**	**Sulfafurazole**	-5.77	2.02
**75**	**Sulfalene**	-6.17	2.01
**76**	**Sulfamethazine**	-6.56	0.88
**77**	**Sulfamethoxazole**	-5.69	1.22
**78**	**Sulfamethoxypyridazine**	-5.98	1.72
**79**	**Sulfametoxydiazine**	-6.05	1.35
**80**	**Sulfamoxole**	-5.95	0.96
**81**	**Sulfanitran**	-6.74	1.71
**82**	**Sulfisomidine**	-6.09	1.81
**83**	**Terephtyl**	-9.95	1.96
**Tetracyclin**			
**84**	**Chlortetracylcin**	-7.24	1.28
**85**	**Democyclin**	-7.67	1.51
**86**	**Doxycycline**	-7.95	2.01
**87**	**Eravacycline**	-8.84	1.89
**88**	**Lymecycline**	-9.13	1.35
**89**	**Meclocycline**	-7.13	1.87
**90**	**Metacycline**	-7.28	1.64
**91**	**Minocycline**	-7.57	0.81
**92**	**Omadacycline**	-8.67	1.31
**93**	**Oxitetracyclin**	-7.32	1.77
**94**	**Rolitetracycline**	-8.78	1.78
**95**	**Sarecycline**	-8.36	1.36
**96**	**Tetracycline**	-7.38	0.97
**97**	**Tigecycline**	-8.75	1.61
**98**	**Penimepicycline**	-7.55	1.78
**Quinolones**			
**99**	**Cinoxacin**	-5.90	1.41
**100**	**Flumequine**	-5.85	1.36
**101**	**Nalidixic acid**	-5.81	1.32
**102**	**Oxolinic acid**	-5.99	1.40
**103**	**Piromidic acid**	-6.29	2.15
**104**	**Rosoxacin**	-6.32	0.58
**105**	**Ciprofloxacin**	-6.29	0.77
**106**	**Danofloxacin**	-6.83	1.59
**107**	**Difloxacin**	-7.27	1.03
**108**	**Enoxacin**	-6.37	1.06

**109**	**Enrofloxacin**	-7.09	1.20
**110**	**Fleroxacin**	-6.99	1.18
**111**	**Lomefloxacin**	-6.76	1.76
**112**	**Nadifloxacin**	-6.90	1.64
**113**	**Norfloxacin**	-6.46	1.26
**114**	**Ofloxacin**	-7.00	1.80
**115**	**Pefloxacin**	-6.67	1.59
**116**	**Rufloxacin**	-6.87	1.39
**117**	**Balofloxacin**	-7.24	1.47
**118**	**Grepafloxacin**	-6.90	1.26
**119**	**Ibafloxacin**	-6.14	1.30
**120**	**Levofloxacin**	-6.99	1.41
**121**	**Marbofloxacin**	-6.84	1.24
**122**	**Orbifloxacin**	-6.98	1.61
**123**	**Pazufloxacin**	-6.25	1.18
**124**	**Sarafloxacin**	-6.88	1.80
**125**	**Sparfloxacin**	-7.31	1.96
**126**	**Temafloxacin**	-7.32	0.99
**127**	**Tosufloxacin**	-6.80	1.52
**128**	**Besifloxacin**	-7.26	1.27
**129**	**Clinafloxacin**	-6.63	1.75
**130**	**Delafloxacin**	-7.13	1.88
**131**	**Gatifloxacin**	-6.87	1.60
**132**	**Gemifloxacin**	-7.62	1.24
**133**	**Moxifloxacin**	-7.46	1.87
**134**	**Ozenoxacin**	-7.13	0.79
**135**	**Prulifloxacin**	-8.08	1.60
**136**	**Sitafloxacin**	-7.23	1.13
**137**	**Trovafloxacin**	-7.30	1.38
**138**	**Fuscidic acid**	-8.25	1.92
**139**	**Co-crystallized inhibitor (EVP)**	-10.51	1.42

Materials and Methods

SI 1: Molecular dynamics simulations

The MD simulations were carried out using Desmond simulation package of Schrödinger LLC.[1] The NPT ensemble with the temperature 300 K and a pressure 1 bar was applied in all runs. The simulation length was 200 ns with a relaxation time 1 ps for the ligands. The OPLS3 force field parameters were used in all simulations.[2] The cutoff radius in Coulomb interactions was 9.0 Å. The orthorhombic periodic box boundaries were set 10 Å away from the protein atoms. The water molecules were explicitly described using the transferable intermolecular potential with three points (TIP3P) model.[3, 4] Salt concentration set to 0.15 M NaCl and was built using the System Builder utility of Desmond.[5] The Martyna−Tuckerman−Klein chain coupling scheme with a coupling constant of 2.0 ps was used for the pressure control and the Nosé−Hoover chain coupling scheme for the temperature control.[6, 7] Nonbonded forces were calculated using a RESPA integrator where the short-range forces were updated every step and the long-range forces were updated every three steps. The trajectories were saved at 20 ns intervals for analysis. The behavior and interactions between the ligands and protein were analyzed using the Simulation Interaction Diagram tool implemented in Desmond MD package. The stability of MD simulations was monitored by looking on the RMSD of the ligand and protein atom positions in time.

SI 2: MD trajectory analysis and prime MM-GBSA calculations

Simulation interactions diagram panel of Maestro software was used to monitoring interactions contribution in the ligand-protein stability. The molecular mechanics generalized born/solvent accessibility (MM – GBSA) was performed to calculate the ligand binding free energies and ligand strain energies for docked compounds over the last 50 ns with thermal_mmgbsa.py python script provided by Schrodinger which takes a Desmond trajectory file, splits it into individual snapshots, runs the MM-GBSA calculations on each frame, and outputs the average computed binding energy. A 1000 frame were extracted from the last 50 ns were subject for MM-GBSA binding energy.

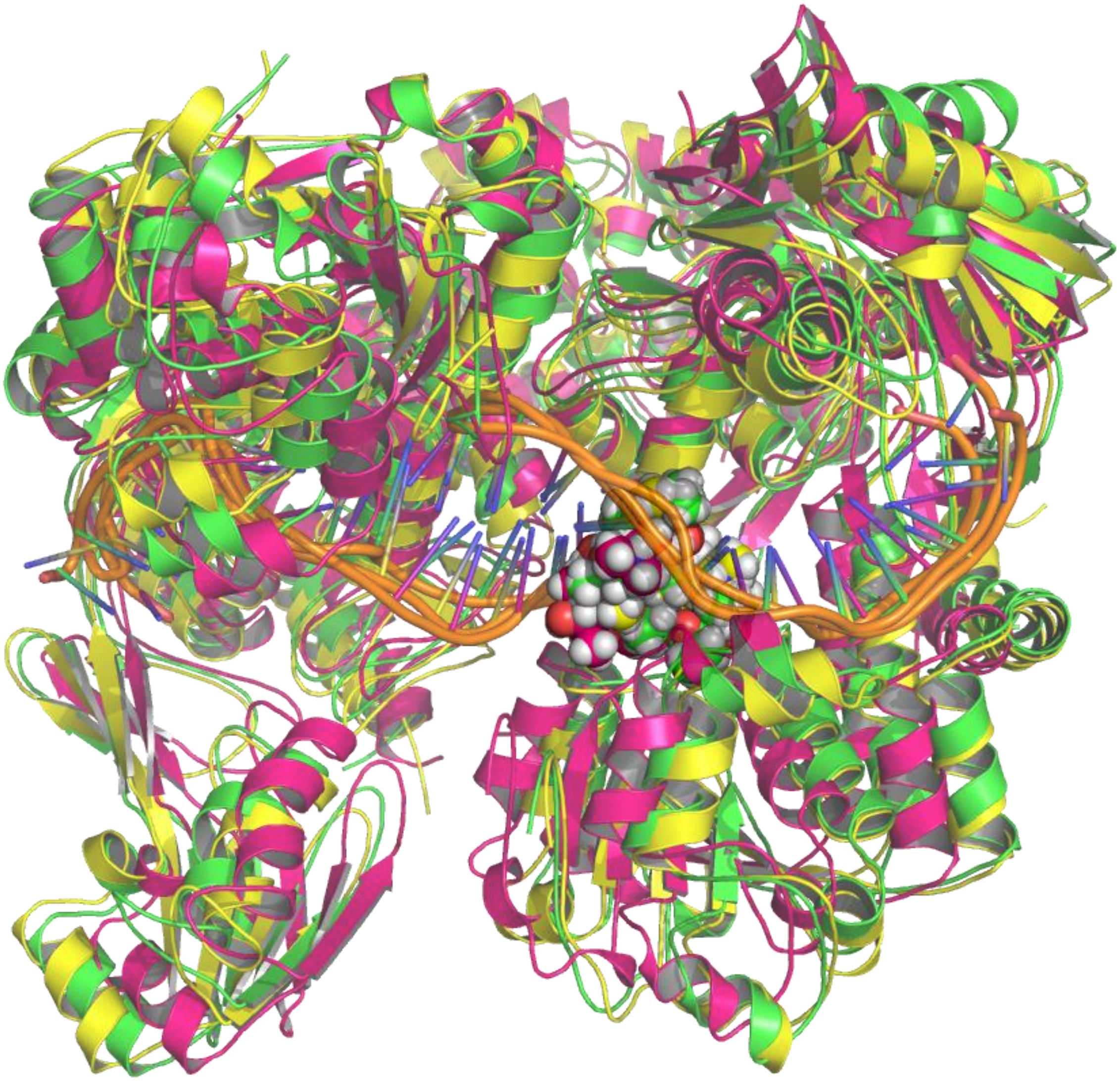

Figure SI 1: Snapshot of **AZ** at 0 ns (red), 100 ns (yellow), and 200 ns (green).

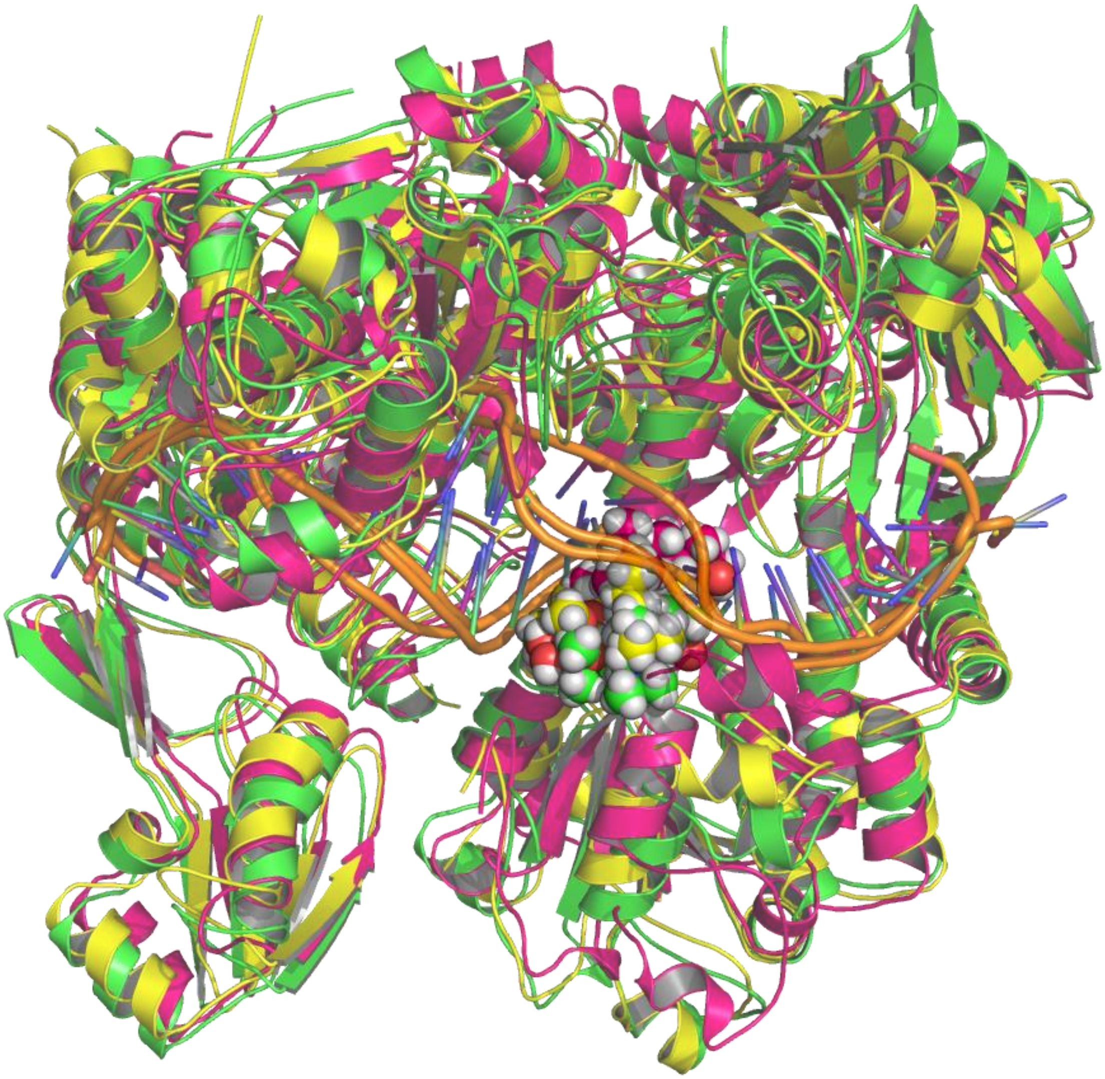

Figure SI 2: Snapshot of **CL** at 0 ns (red), 100 ns (yellow), and 200 ns (green).

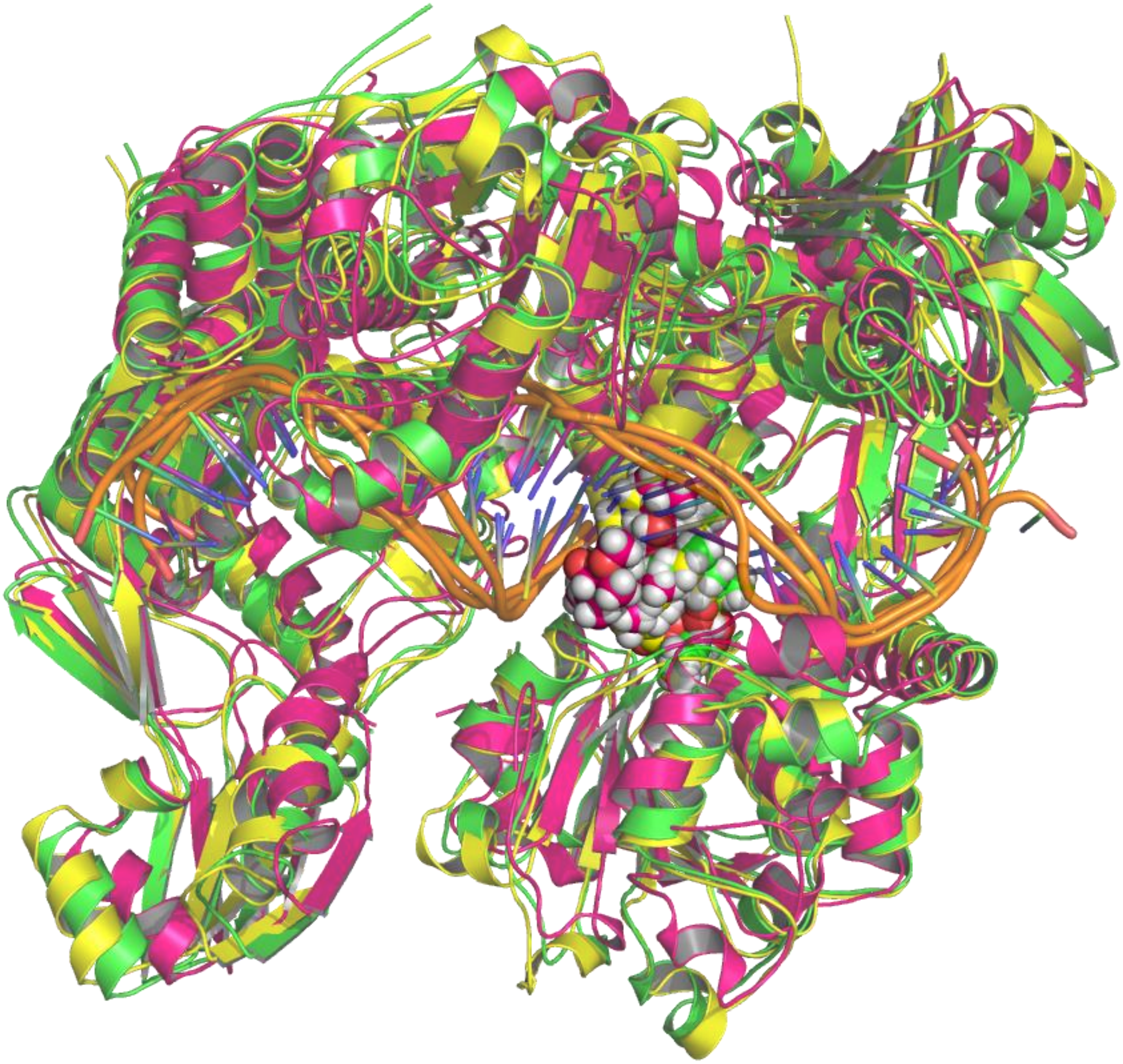

Figure SI 3: Snapshot of **ER** at 0 ns (red), 100 ns (yellow), and 200 ns (green).

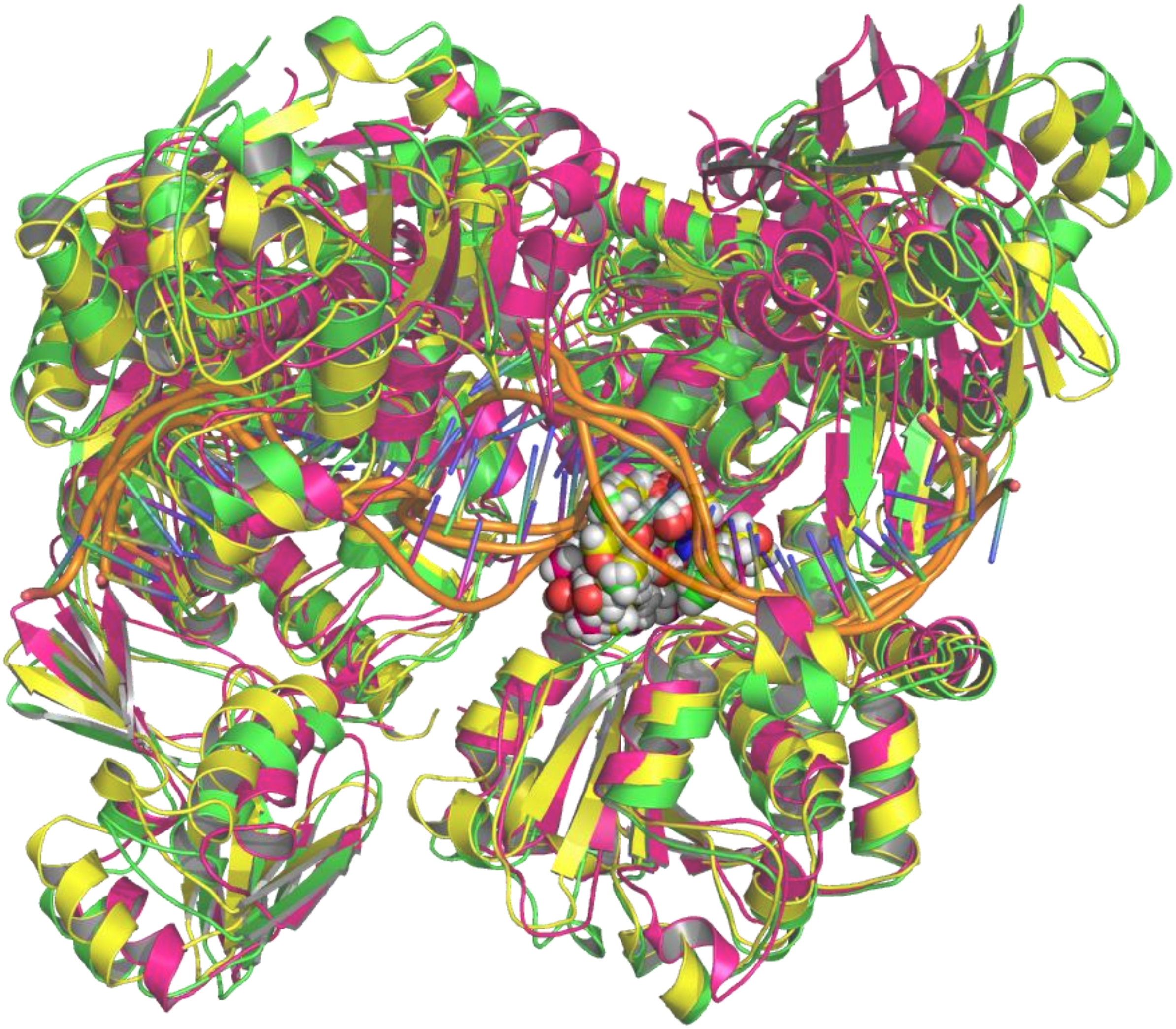

Figure SI 4: Snapshot of **RO** at 0 ns (red), 100 ns (yellow), and 200 ns (green).

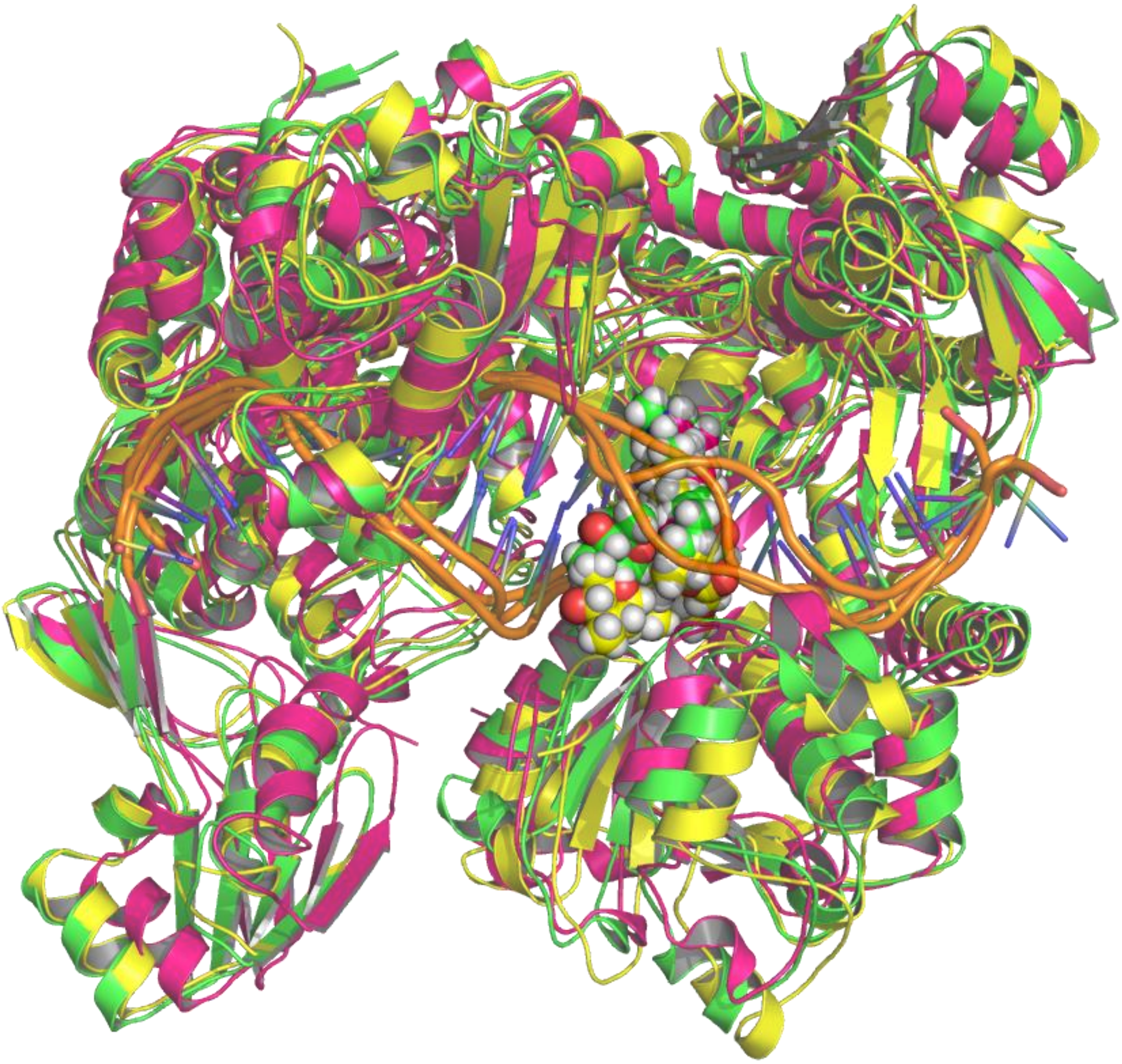

Figure SI 5: Snapshot of **SP** at 0 ns (red), 100 ns (yellow), and 200 ns (green).

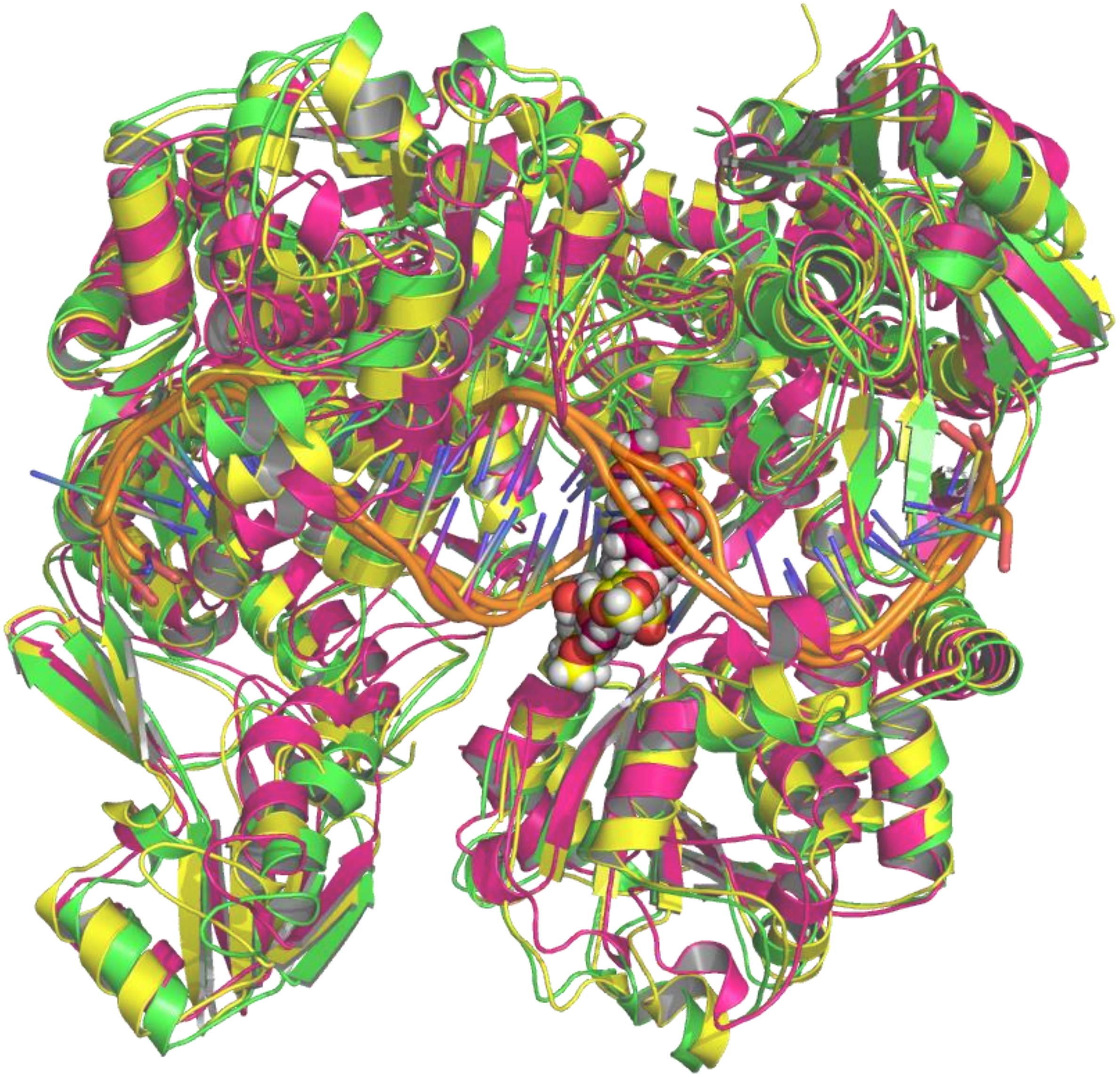

Figure SI 6: Snapshot of **EVP** at 0 ns (red), 100 ns (yellow), and 200 ns (green).

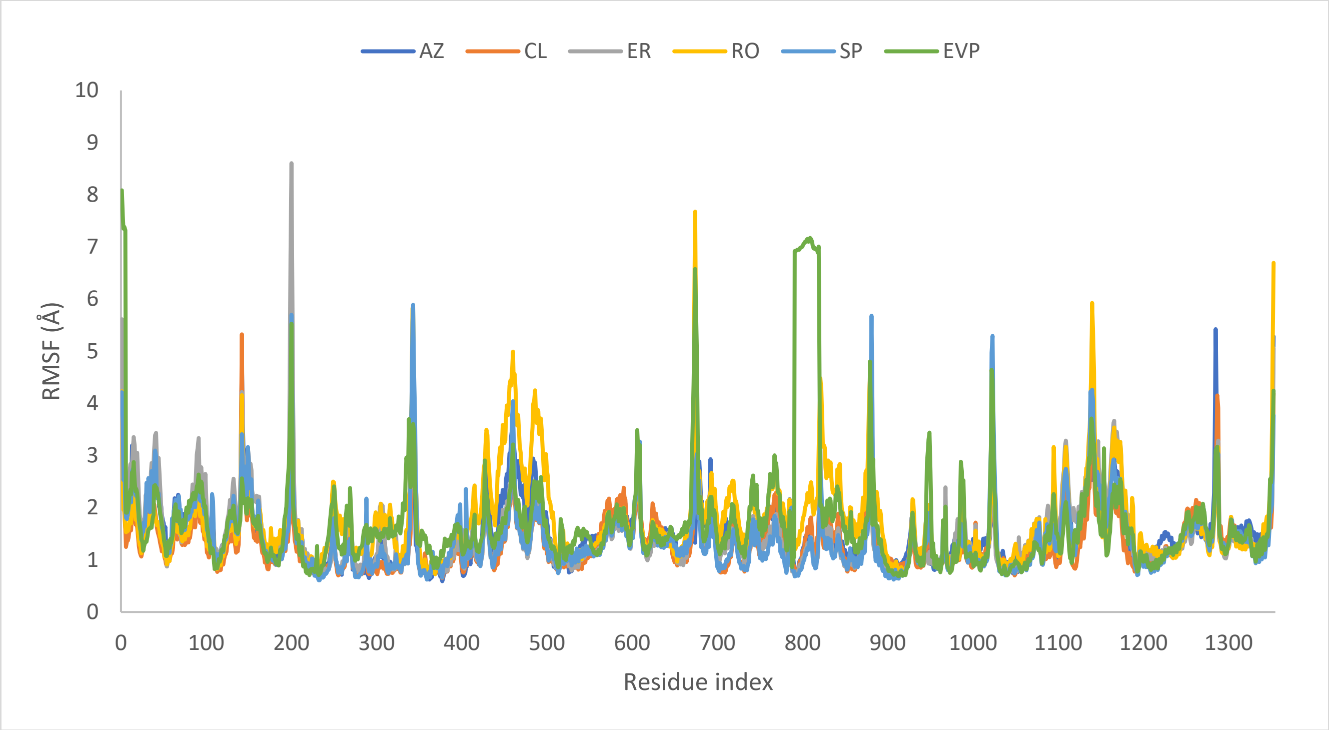

Figure SI 7: The RMSF of the proteins Cα during the simaultion time for compounds **AZ**, **CL**, **ER**, **RO**, **SP**, and **EVP**.

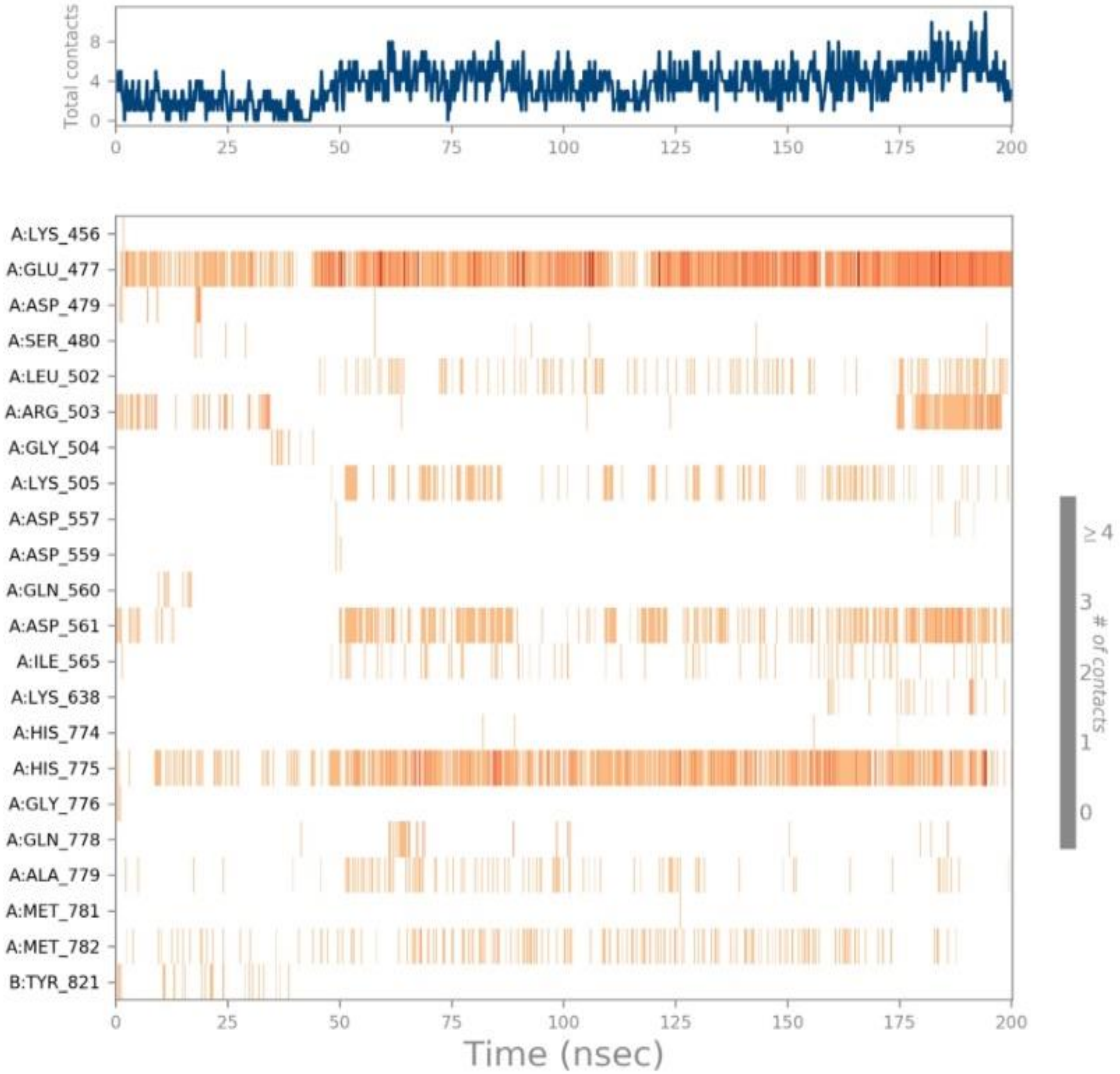

Figure SI 8: Heat map describing the protein-ligand interactions regarding the simulation time of 200 ns for **AZ**-3QX3.

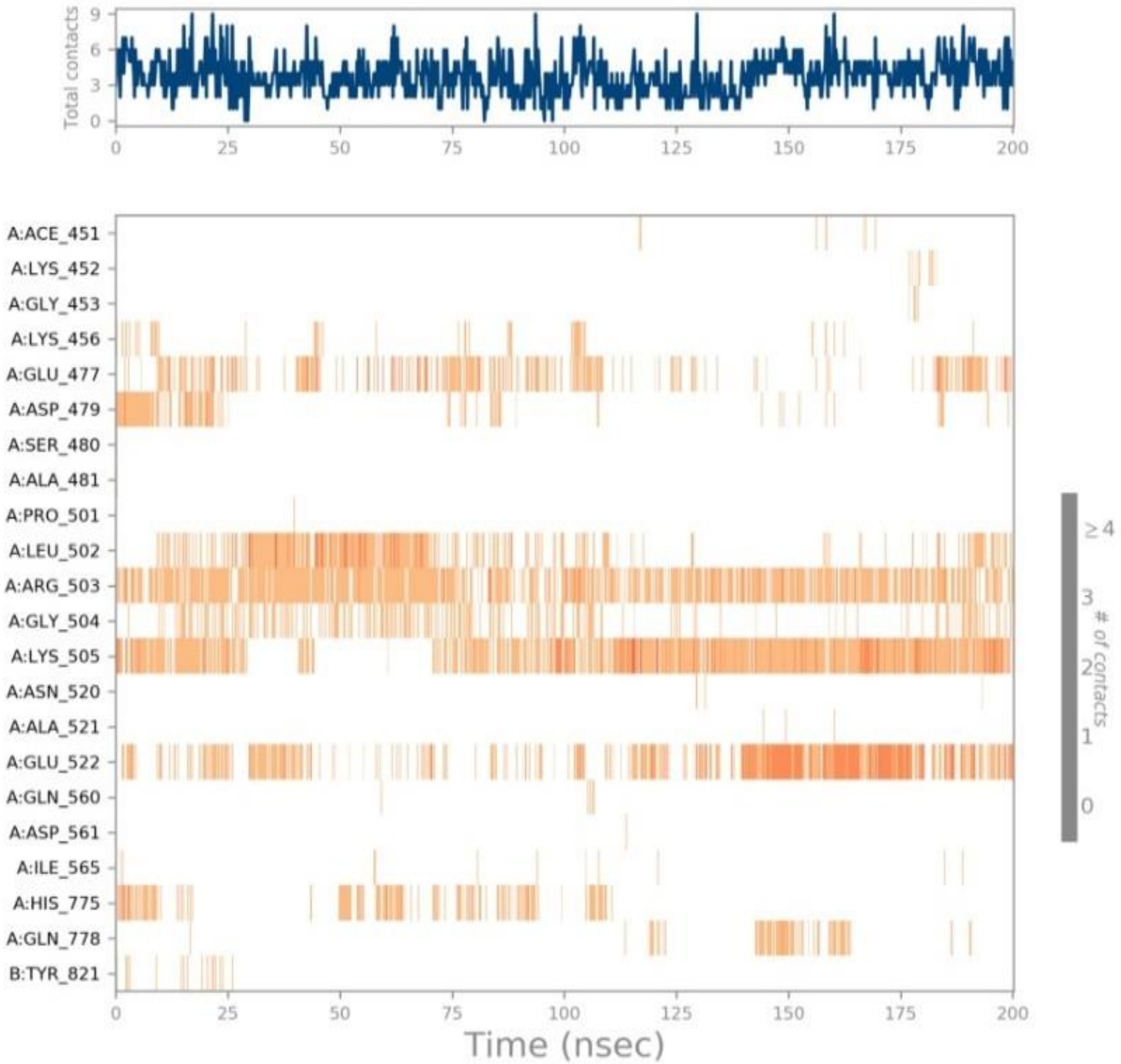

Figure SI 9: Heat map describing the protein-ligand interactions regarding the simulation time of 200 ns for **CL**-3QX3.

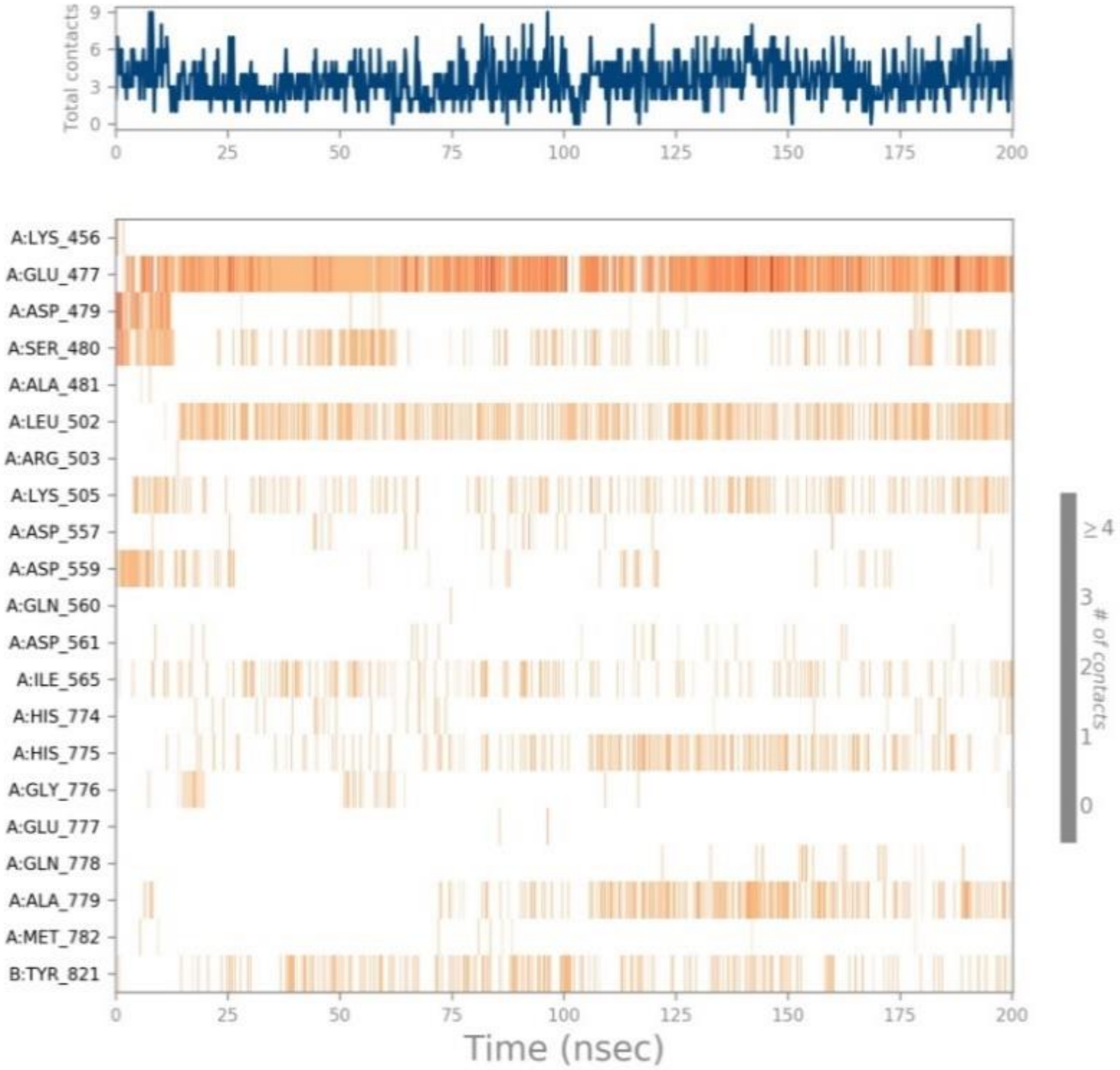

Figure SI 10: Heat map describing the protein-ligand interactions regarding the simulation time of 200 ns for **ER**-3QX3.

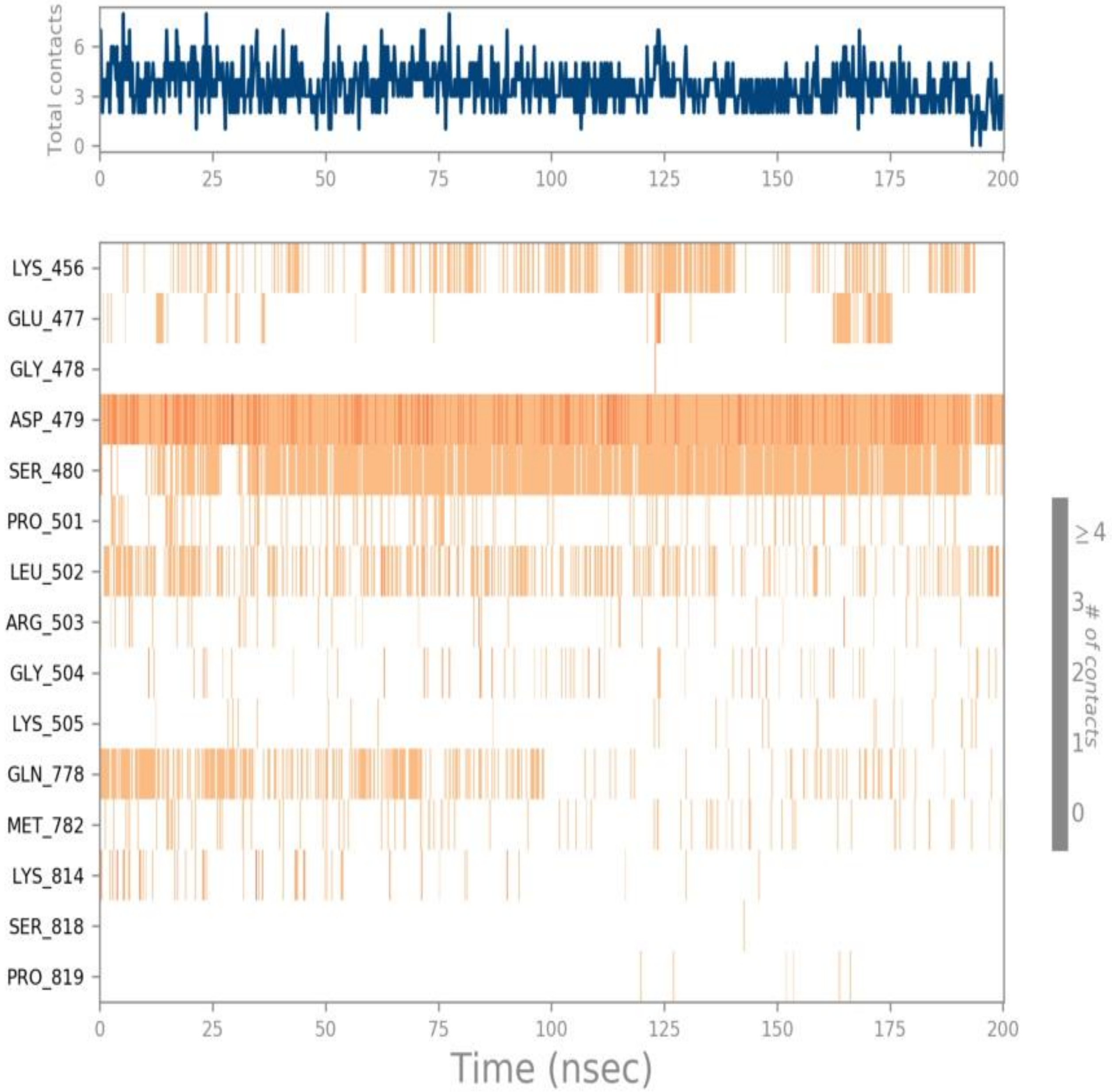

Figure SI 11: Heat map describing the protein-ligand interactions regarding the simulation time of 200 ns for **EVP**-3QX3.

References

1. Release, S., 3: Desmond molecular dynamics system, DE Shaw research, New York, NY, 2017. Maestro-Desmond Interoperability Tools, Schrödinger, New York, NY **2017**.

2. Harder, E.; Damm, W.; Maple, J.; Wu, C.; Reboul, M.; Xiang, J. Y.; Wang, L.; Lupyan, D.; Dahlgren, M. K.; Knight, J. L., OPLS3: a force field providing broad coverage of drug-like small molecules and proteins. Journal of chemical theory and computation **2016,** 12 (1), 281-296.

3. Jorgensen, W. L.; Chandrasekhar, J.; Madura, J. D.; Impey, R. W.; Klein, M. L., Comparison of simple potential functions for simulating liquid water. The Journal of chemical physics **1983,** 79 (2), 926-935.

4. Neria, E.; Fischer, S.; Karplus, M., Simulation of activation free energies in molecular systems. The Journal of chemical physics **1996,** 105 (5), 1902-1921.

5. Manual, D. U., Desmond2. 2. **2009**.

6. Martyna, G. J.; Klein, M. L.; Tuckerman, M., Nosé–Hoover chains: The canonical ensemble via continuous dynamics. The Journal of chemical physics **1992,** 97 (4), 2635-2643.

7. Martyna, G. J.; Tobias, D. J.; Klein, M. L., Constant pressure molecular dynamics algorithms. The Journal of chemical physics **1994,** 101 (5), 4177-4189.
